# Neuro‐Cells therapy improves motor outcomes and suppresses inflammation during experimental syndrome of amyotrophic lateral sclerosis in mice

**DOI:** 10.1111/cns.13280

**Published:** 2019-12-23

**Authors:** Johannes P.J.M. de Munter, Igor Shafarevich, Alexei Liundup, Dmitrii Pavlov, Erik Ch Wolters, Anna Gorlova, Ekaterina Veniaminova, Aleksei Umriukhin, Allan Kalueff, Andrei Svistunov, Boris W. Kramer, Klaus‐Peter Lesch, Tatyana Strekalova

**Affiliations:** ^1^ Department of Psychiatry and Neuropsychology School for Mental Health and Neuroscience Maastricht University Maastricht The Netherlands; ^2^ Laboratory of Psychiatric Neurobiology Institute of Molecular Medicine and Department of Normal Physiology Sechenov First Moscow State Medical University Moscow Russia; ^3^ Institute of Regenerative Medicine Sechenov First Moscow State Medical University Moscow Russia; ^4^ Laboratory of Cognitive Dysfunctions Institute of General Pathology and Pathophysiology Moscow Russia; ^5^ Faculty of Biology Ural Federal University Ekaterinburg Russia; ^6^ School of Pharmacy Southwest University Chongqing China; ^7^ Department of Pediatrics University Medical Center (MUCM) Maastricht The Netherlands; ^8^ Division of Molecular Psychiatry Center of Mental Health University of Würzburg Würzburg Germany

**Keywords:** amyotrophic lateral sclerosis (ALS), fused in sarcoma (FUS) protein, glycogen‐synthase kinase‐3ß (GSK‐3ß), microglia activation, mouse, stem cell therapy, superoxide dismutase‐1 (SOD‐1) G93A mice

## Abstract

**Aims:**

Mutations in DNA/RNA‐binding factor (fused‐in‐sarcoma) FUS and superoxide dismutase‐1 (SOD‐1) cause amyotrophic lateral sclerosis (ALS). They were reproduced in SOD‐1‐G93A (SOD‐1) and new FUS[1‐359]‐transgenic (FUS‐tg) mice, where inflammation contributes to disease progression. The effects of standard disease therapy and anti‐inflammatory treatments were investigated using these mutants.

**Methods:**

FUS‐tg mice or controls received either vehicle, or standard ALS treatment riluzole (8 mg/kg/day), or anti‐inflammatory drug a selective blocker of cyclooxygenase‐2 celecoxib (30 mg/kg/day) for six weeks, or a single intracerebroventricular (i.c.v.) infusion of Neuro‐Cells (a preparation of 1.39 × 10^6^ mesenchymal and hemopoietic human stem cells, containing 5 × 10^5^ of CD34^+^ cells), which showed anti‐inflammatory properties. SOD‐1 mice received i.c.v.‐administration of Neuro‐Cells or vehicle.

**Results:**

All FUS‐tg‐treated animals displayed less marked reductions in weight gain, food/water intake, and motor deficits than FUS‐tg‐vehicle‐treated mice. Neuro‐Cell‐treated mutants had reduced muscle atrophy and lumbar motor neuron degeneration. This group but not celecoxib‐FUS‐tg‐treated mice had ameliorated motor performance and lumbar expression of microglial activation marker, ionized calcium‐binding adapter molecule‐1 (Iba‐1), and glycogen‐synthase‐kinase‐3ß (GSK‐3ß). The Neuro‐Cells‐treated‐SOD‐1 mice showed better motor functions than vehicle‐treated‐SOD‐1 group.

**Conclusion:**

The neuropathology in FUS‐tg mice is sensitive to standard ALS treatments and Neuro‐Cells infusion. The latter improves motor outcomes in two ALS models possibly by suppressing microglial activation.

## 1 INTRODUCTION

Amyotrophic lateral sclerosis (ALS) is a neurodegenerative disease characterized by progressive degeneration of lower motor neurons, as well as neurons in the cortex and brainstem, which leads to paralysis and premature death.^1^ The etiology of ALS remains unclear both in sporadic cases (90%) and in the familial forms of ALS.^1^, ^2^ Among the known genetic causes that give rise to ALS, the mutation of the fused in sarcoma protein (FUS) is the second most frequent among the familial forms of ALS.^2^, ^3^, ^4^

Mutations of FUS gene were thought to cause synaptic dysfunction and pathological protein aggregation,^5^ which were felt to be key events leading to neuronal degeneration.^6^ However, the most recent studies have revealed that the expression of mutant FUS leads to stress‐mediated induction of chaperones, decreased expression of ion channels and transporters essential for synaptic function, and reduced synaptic activity without the loss of nuclear FUS or its cytoplasmic aggregation.^7^ The nuclear effects of FUS also seem to result in impairment of the function of paraspeckles, granules in the nuclear interchromatin space that are assembled on a scaffold long noncoding RNA (lncRNA) NEAT1.^8^, ^9^, ^10^ This results in aberrant microRNA biogenesis, apoptotic processes, oxidative stress, and mitochondrial dysfunction contributing to neurodegenerative processes.^9^, ^10^ Indeed, the latest studies using human fibroblast cell lines expressing mutant FUS from ALS patients and postmortem tissue have identified the accumulation of dysfunctional paraspeckles associated with abnormal NEAT1 expression as an important feature of FUS‐associated ALS pathology.^11^ NEAT1 was recently reported to promote inflammation via stimulating interleukin‐1β production and pyroptosis and activating macrophages.^12^, ^13^

Regardless of whether the disease is associated with FUS gene mutations or related to other factors, its pathological mechanisms are associated with neuroinflammation.^1^, ^2^, ^4^, ^14^ In particular, the activation of microglia and astrocytes is considered to be a hallmark of the disease and is accompanied by elevated pro‐inflammatory cytokine concentrations in the brain, blood, and cerebrospinal fluid.^14^, ^15^, ^16^ For example, a recent clinical study reported high blood concentrations of pro‐inflammatory cytokines and related proteins in ALS patients, including interleukin‐1β (IL‐1β), interleukin 6 (IL‐6), interleukin‐8 (IL‐8), tumor necrosis factor (TNF), and TNF receptor‐1.^17^ It has been suggested that microglial inflammatory processes might be a key early event that contribute to neurodegeneration and play a dynamic role in the pathogenesis of the disease.^14^, ^16^ Cerebrospinal fluid from ALS patients induces marked microglial activation, and upregulation of the pro‐inflammatory cytokines and factors including IL‐6, TNF, cyclooxygenase‐2 (COX‐2), and prostaglandin E2 (PGE2), accompanied by a downregulation of trophic factors.^15^, ^18^

Despite the evidence that the level of inflammation is critical in ALS, clinical studies with compounds that target inflammatory mechanisms and associated cascades, including the TNF inhibitor thalidomide, nonsteroid anti‐inflammatory drugs (NSAIDs), a selective COX‐2 inhibitor celecoxib, corticosteroids, cyclophosphamide, cyclosporine, cytochrome C inhibitors, and caspase‐reducing drugs have all failed to induce significant improvement of the ALS pathology.^19^, ^20^, ^21^ Thus, while the therapeutic niche for anti‐inflammatory treatment of the ALS is strongly implicated by clinical and preclinical studies, current literature lacks any clear examples of positive results.

In the present study, we sought to test the effects of a stem cell therapy “Neuro‐Cells” with anti‐inflammatory actions^22^, ^23^ on experimental models of ALS in mice. Therefore, we have employed a FUS[1‐359]‐tg mouse model^9^ according to the guidelines set by Ludolph et al, 2010, for ALS preclinical studies^24^ that have recommended the use of other ALS models apart from the SOD‐1 mouse “gold standard”.^25^ The pattern of pathology in FUS[1‐359]‐tg line recapitulates key features of human ALS, including motor neuron degeneration and microgliosis in the brainstem and spinal cord, muscle atrophy, paralysis, microglial activation, and elevated levels of pro‐inflammatory cytokines in the CNS and blood.^9^, ^26^, ^27^, ^28^ The model permits the impact of therapy on ALS‐related changes to be evaluated including basic physiological and motor functions, as well as evaluation of the expression of pro‐inflammatory and degeneration markers such as Iba‐1, GSK‐3ß, IL‐1ß, and IL‐6 in the lumbar spinal cord and blood levels of IL‐1ß and IL‐6. In the FUS model, the effect of the standard therapies has not been studied. We therefore chose to compare them to Neuro‐Cells. In addition, we examined the effects of Neuro‐Cells administration on motor function in the SOD‐1 mouse line, a well‐established model of ALS (G93A line;^25^).

Here, we gave in both models a single inracerebroventricular (i.c.v.) infusion of a preparation of nonmanipulated human stem cells “Neuro‐Cells” obtained from bone marrow, which are a combination of both mesenchymal stem cells (MSCs) and hemopoietic stem cells (HSCs). As both MSCs and HSCs have been shown to have the ability to differentiate into a spectrum of adult cell populations, many studies have sought to examine either the combined or individual contributions to repair in vivo in models of injury and potentially capitalize on the relationship between the two cell populations that is known to exist.^29^, ^30^ As for instance, MSCs were shown to act as a feeder layer maintaining HSCs in an undifferentiated state. If HSCs are allowed to differentiate during their expansion, it increases the process of cell aging and death.^31^

In spinal injury models, the engraftment of CD34^+^ human HSCs produces neurons efficiently in the regenerating chicken embryo spinal cord^32^ and the use of MSCs to form guiding strands in the injured spinal cord promotes recovery.^33^ A direct comparison has been made between human mononuclear cell preparations (a mixture of HSCs and MSCs) and culture‐expanded MSCs transplanted into a spinal cord injury model in rats without differences with regard to graft efficiency, spinal cord tissue sparing, or glial scar reduction.^34^

In view of these results, we found that an injection of a mixture of MSC and HSC, to maximize the potential benefit of these populations, had an anti‐inflammatory effect.^23^ The intrathecal administration of the MSC/HSC preparation, containing 4 × 10^5^ of CD34^+^ cells into rats that had been subjected to a spinal cord injury, was shown to improve functional outcomes and decrease peripheral concentrations of pro‐inflammatory cytokines (TNF, IL‐1ß, and IL‐6) in the cerebrospinal fluid.^23^ Previous studies have reported beneficial effects of stem cell therapy in animal models of ALS^35^, ^36^, ^37^; the majority of these studies employed manipulated MCSs,^35^, ^38^, ^39^, ^40^, ^41^, ^42^ while the use of unmanipulated stem cells and preparations containing HSCs and MSCs has been shown to increase the therapeutic activity of stem cell therapy.^41^, ^42^, ^43^ Thus, we hypothesized that this treatment might also ameliorate ALS‐like pathology owing to the anti‐inflammatory effects of Neuro‐Cells. Here, we sought compare the effects of Neuro‐Cells to the current anti‐ALS therapy riluzole^44^ and the classic anti‐inflammatory compound celecoxib^45^, ^46^ in the new FUS‐tg model.

## 2 MATERIAL AND METHODS

Details on animals and all experimental procedures can be found in Data S1.

### 2.1 Animals and human endpoint

FUS‐tg, SOD‐1 male mice and their wild‐type littermates (WT) were provided by the FDA‐certified IPAC RAS facilities and Charles River provider, respectively (http://www.ipac.ac. ru/index.html and http://www.spf-animals.ru/about/providers/animals). Mice were single housed under standard conditions and reversed lighting. Experimental procedures were set up in accordance with a Directive 2010/63/EU and approved by the local veterinarian Committee for Bioethics of IPAC RAS (N19‐16.06.2017) and MSMU (22/10/17‐MSMU‐35). Bone marrow collection from healthy volunteers was done under GMP license (Neuroplast BV Farmatec The Netherlands) and approved by Ethical Committee of MUMC, Maastricht University (iCell1 METC MUMC and iCell2 METC Zuyderland Zuid). All efforts were undertaken to ensure compliance with above‐mentioned regulations concerning human endpoint in animal research.

### 2.2 Study design

Experimental designs were based on reported patterns of pathology in employed models. ALS pathology in 12‐week‐old FUS‐tg mutants is characterized by rapid, progressive motor neuron degeneration within 2 weeks,^9^ while SOD‐1 mouse line (G93A mutants) has more gradually developing ALS pathology displaying its first signs by the age of about 16 weeks 25 progressing to the human endpoint within 4‐6 weeks.^47^, ^48^ We devoted our efforts to ensure compliance with above‐mentioned observations concerning human endpoint in animal research.

**Figure 1:**
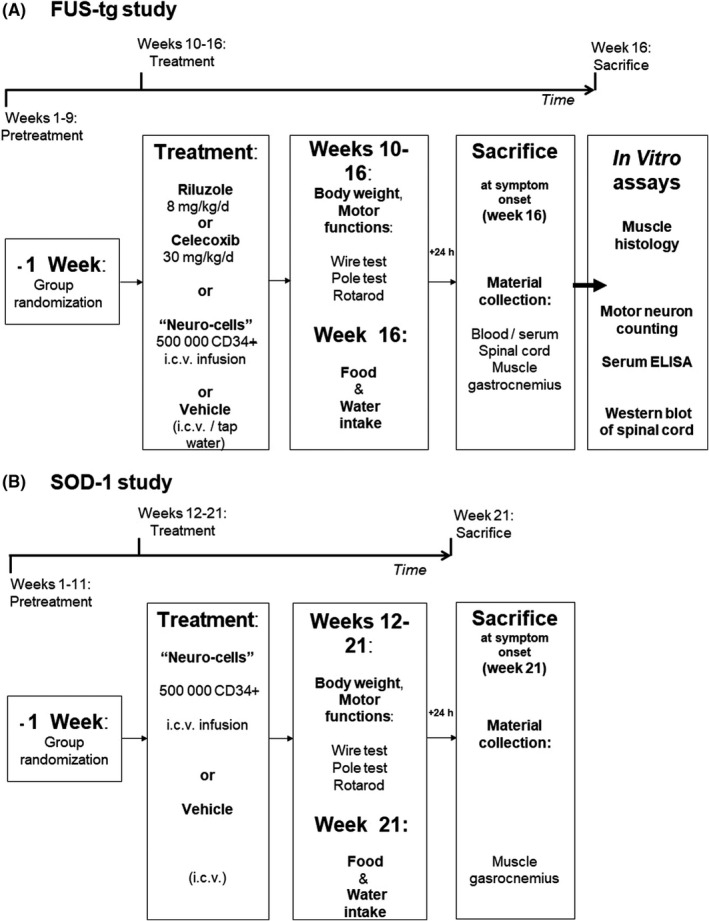
Experiment design of study on (A) FUS‐tg mice and (B) SOD‐1 mice

At the age of 7‐8 weeks, FUS‐tg animals and wild‐type (WT) controls were studied in a cat‐walk, grip test, weighed (*data not shown*), and assigned to groups. At the age of nine weeks, FUS‐tg and WT mice received (a) regular tap water, or (b) riluzole (Ril; 8 mg/kg/day, via drinking water), or (c) or celecoxib (Cel, 30 mg/kg/day) via food pellets, or (d) single i.c.v. administration of Neuro‐Cells (NC, 500 000‐CD34^+^ in 10 μL of Ringer Lactate buffer), or (d) i.c.v. administration of Ringer Lactate buffer (Figure 1A). Doses of pharmaca were selected as described previously (49‐51; see below). Stereotaxic surgery was adapted from previously reported procedure.^52^ During the following six weeks, all mice were weekly weighed and studied in the rotarod, pole, and wire tests for motor function, dosing with pharmaca was continued. As postsurgery physiological parameters of wild‐type mice that received either tap water or i.c.v. vehicle injection were similar (Figure S1), these two groups were merged into a vehicle‐treated (Veh) group for subsequent analysis. On Week 6, at the age of fifteen weeks, mice were investigated for food and water intake and then sacrificed 24 hours thereafter. Mice were either perfused with NaCl or 4%‐paraformaldehyde; muscle gastrocnemius, blood, and spinal cord were harvested for weighing, Western blot, ELISA assays, and histology. Since Neuro‐Cells‐treated animals showed greater functional improvement than other groups, these mice and mutants subjected to the i.c.v. vehicle infusion were studied in all in vitro assays including muscle atrophy and motor neuron counts in the lumbar part of the spinal cord. The concentrations of IL‐1ß and IL‐6 in blood serum and protein expression of IL‐1ß, IL‐6, Iba‐1, and GSK‐3ß in the lumbar part of the spinal cord of mice treated with either vehicle, Neuro‐Cells, or celecoxib were measured by ELISA and Western blot assays. Pharmaca outline and genotype were double blind for all experimenters. Numbers of animals used are indicated in Figure legends.

In additional experiment, SOD‐1 male mice (G93A line) at age 12 weeks were subjected to the i.c.v. infusion of the Neuro‐Cells from the batch used in FUS‐tg animals (SOD‐1‐NC group, n = 7) or to the i.c.v. vehicle administration (SOD‐1‐Veh group, n = 5; Figure 1B). Mice were weekly weighed and tested for motor functions for nine weeks as in a previous study and sacrificed at age of 21 weeks, at the onset of ALS‐like syndrome in this model.^25^, ^47^, ^48^, ^49^

### 2.3 Functional readouts

Weekly measured body weights of mice were normalized to their baseline values recorded at week 1 (Figure 1). Body weights during weeks 1‐6 and weight gain between these weeks were normalized to the mean values of wild‐type mice treated with vehicle (WT‐Veh). On the 6th week, 24‐hour food intake and 12‐hour water intake were evaluated as described elsewhere.^51^

#### 2.3.1 Wire test

Mice were allowed to grip a horizontal wire (diameter 0.3 cm, height above the surface 60 cm) for 180s. The latency of falling and the number and percent of mice with falling events (latency < 20s) were recorded as described elsewhere.^53^

#### 2.3.2 Pole test

Mice were placed on a top of the vertical bar (diameter 1.1 cm, height 60 cm) and allowed to climb down to a horizontal surface. The latency of descending the bar and the number and percent of mice with sliding events (latency to descend < 50s) were scored as described elsewhere.^53^

#### 2.3.3 Rotarod

Mice were placed on constantly rotting rod of rotarod (Columbus Instruments, Columbus, OH, USA; speed 10 rpm) for 600s. Latency to fall and the number and percent of mice with falling events (latency < 200s) were registered in three runs as described elsewhere.^53^

### 2.4 Administration of drugs

In the study with FUS‐tg mice, potential effects of Neuro‐Cells were compared to effects of celecoxib, NSAID, and a COX‐2 inhibitor, that was used as a classic anti‐inflammatory compound,^45^, ^46^, ^50^ and of riluzole (2‐amino‐6‐trifluoromethoxy‐benzothiazole), a standard ALS treatment that can slow down the fatal disease progress by 2‐3 months^44^ and frequently serves as a reference drug in translational studies with ALS.^47^, ^49^ Riluzole tablets (Sandoz, Almere, Netherlands) were crushed and dissolved in tap water, and its concentration was adjusted to the dosage of 8 mg/kg/day and daily water intake in CD1 mice.^47^, ^49^ elecoxib‐containing food pellets were produced as described elsewhere,^51^ and drug concentration was adjusted to the dosage of 30 mg/kg/day and daily diet consumption.

### 2.5 Generation, properties, and intracerebroventricular infusion of Neuro‐Cells

Neuro‐Cells, a preparation of human bone marrow‐derived HSCs and MSCs, was provided by Neuroplast BV (Maastricht, Netherlands). The Neuro‐Cell preparation comprised 1.39 × 10^6^ MSCs and HSCs, containing 5 × 10^5^ CD34^+^ cells in 10 μL. The expression profile of MSC markers was overlapping; in the total cell preparation 85.6% were CD105^+^, 13% were CD90^+^, 7% were CD271^+^, and 4% were CD73^+^ in single FACs staining. Prior to infusion, cells were resuspended and checked for vitality (see Data S1 and Table S2). Animals were anesthetized by halothane (Halothane TM; Willy Rusch, Boblingen, Germany) and immobilized in a stereotaxic frame (World Precision Instruments, Sarasota, TX, USA) for unilateral i.c.v. infusion via a hole made in a scull of mice as described elsewhere.^52^ All preparations of Neuro‐Cells were from the same stock sample. They were arranged *ex temporo*; after thawing, cell counting was performed using the “Countess II FL Automated Cell Counter” (Thermo Fisher Scientific AMQAF1000, Toronto, ON, Canada) showing 62%‐68% cell vitality (see Data S1). Subsequent flow cytometry was done, and HSC numbers were adjusted for injection.

Pilot studies carried out to determine the distribution of infused Neuro‐Cells and optimize the protocols for the i.c.v. administration, suggested their presence in the brain and peripheral organs 12 and 24 hours after injection (100, 000 or 250, 000 cells were infused to lateral ventricles in 12 mice; see Data S1). Immunohistochemical study of Neuro‐Cells‐treated mice with human antimitochondrial antibodies was carried out as described elsewhere^54^ and revealed a positive signal in their ventricles, brain tissue, lungs, and spleen, suggesting a wide distribution of Neuro‐Cells in the cerebrospinal fluid and persistent vitality (see Data S1; Figures S1 and S2). These findings are consistent with the results of the pilot study with i.c.v. infusion of bone marrow‐derived mouse stem cells obtained from mutants expressing green fluorescent protein (GFP) (see Data S1; Figure S3,^55^)These experiments showed that i.c.v. administration of Neuro‐Cells to mice at the concentrations 100 000‐500 000 is well tolerated.

### 2.6 Blood and tissue collection

Mice were terminally anesthetized by sodium pentobarbitone and from about a half of them, blood, both gastrocnemius muscles and lumbar parts of the spinal cord were collected as described elsewhere.^56^ Another half was perfused with 4% paraformaldehyde; the lumbar parts of spinal cords and muscle gastrocnemius were dissected as described elsewhere.^56^

### 2.7 Scoring for muscle atrophy

Muscle gastrocnemius was fixed, sectioned, and stained for hematoxylin and eosin. Scoring for atrophy was performed by three independent pathologists, blinded to sample identity using a light microscope (Axiovision 4.3, Zeiss, Berlin, Germany); ranking histograms were generated ranging samples were from 1 to 11 from “moderate” (1) to “severe” (11) atrophy.

### 2.8 Motor neuron counting

Briefly, cross sections were carried out at 50‐μm cuts encompassing L3‐L5 with 250 μm interval and stained with thionine NISSL. Counts were performed by two observers blinded to sample identity using a Zeiss Axoplan2 system (Zeiss, Berlin, Germany).

### 2.9 ELISA of plasma cytokines

Mouse enzyme‐linked immunosorbent assay (ELISA) was performed using MOUSE IL‐1β and IL‐6 ELISA MAX™ Deluxe Sets (BioLegend, San Diego, CA, USA) according to the manufacturer's instructions; protein concentrations were measured using the BCA protein assay kit (Pierce, Rockford, IL, USA) as described elsewhere.^57^

### 2.10 Western blot and protein isolation

Western blot analysis on spinal cord samples and quantification of protein concentration were performed as described elsewhere (58; see Table S3). Relative expression of proteins was calculated in fold changes from levels of β‐tubulin as described elsewhere.^58^ The Western blot images used for quantification are presented in Figure S6 and Appendix S1.

### 2.11 Statistical analysis

GraphPad Prism 6.00 software (San Diego, CA, USA) was used; dependently on groups, multiple group comparisons were analyzed using one‐way, two‐way, and repeated‐measures ANOVA followed by the Tukey's test. Mann‐Whitney test was used for two‐group comparisons. Qualitative data were analyzed by Fisher's exact test. The level of significance was *P* < .05. The results are presented as bars with standard error of means (SEM).

## 3 RESULTS

### 3.1 Physiological parameters in FUS‐tg mice treated with riluzole, celecoxib, or Neuro‐Cells

#### 3.1.1 Body weight, food, and liquid intake

**Figure 2:**
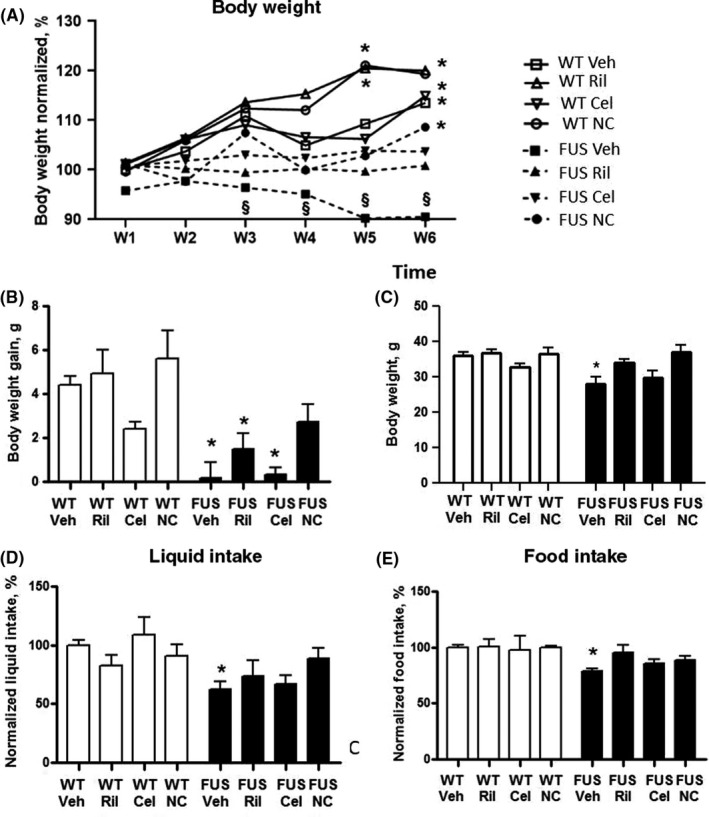
Effects of riluzole, celecoxib, and Neuro‐Cells on physiological parameters of FUS‐tg mice. A, There was a significant increase in body weight in the WT groups, but not in the FUS‐tg‐Veh mice (*P < .05 vs basal values; see the text). FUS‐tg mice‐NC group showed a significant weight gain. B, In comparison with controls, there was significantly lower weight gain in FUS‐tg mice except FUS‐tg‐NC, (*P < .05 vs WT‐Veh group); no such changes were found in the FUS‐tg‐NC group (*P* > .05). C, At Week 6, there was a significant reduction of body weight in FUS‐tg‐Veh group in comparison with wild‐type controls (*P < .05); no such changes were found in mutants that received other treatments (*P* > .05). In comparison with WT‐Veh group, FUS‐tg‐Veh group displayed a significant decrease in (D) liquid intake and (E) food intake (*P < .05), but not other FUS‐tg groups. Each WT‐group was comprised of 8 animals; 7‐11 mice were used in FUS‐tg groups (FUS‐Veh, n = 11; FUS‐Ril, n = 9, FUS‐Cel, n = 7, FUS‐NC, n = 7)

Repeated‐measures ANOVA and post hoc Tukey's tests revealed significant increases in body weight normalized to the basal values in WT‐Veh, WT‐Ril, and WT‐Cel mice from week 1 to week 6 (*F*_3,29_ = 7.86, *P* = .023; Figure 2A). FUS‐tg‐Veh mice displayed a significant decrease in this measure (*F*_3,29_ = 12.76, *P* = .0147). No significant changes in this parameter were found in FUS‐tg mice that received either treatment (*P* > .05) indicating partial rescue effects of all applied treatments on FUS‐tg mice.

Two‐way ANOVA and post hoc Tukey's test revealed a significant effect of genotype on body weight, normalized to the means of control mice at each week of study, at third to sixth weeks (*P* < .05, two‐way ANOVA). This parameter was significantly lower in FUS‐tg‐Veh mice on the third to sixth weeks than in WT‐Veh group (*P* = .031, *P* = .026, *P* = .018 and *P* = .001, respectively; Tukey's test, Figure S5). Such decreases were found in FUS‐tg‐Ril mice on the sixth week (*P* = .043) and in FUS‐tg‐Cel group, on the fifth and sixth weeks (*P* = .025 and *P* = .019, respectively), but not in FUS‐tg‐NC animals.

ANOVA showed significant effects of genotype and treatment for body weight gain (*F*_1,10_ = 27.98, *P* < .0001, and *F*_3,10_ = 3.499, *P* = .0182), but not for their interaction (*F*_3,10_ = 0.7239, *P* = .53499). There was a significant decrease in body weight gain in FUS‐tg‐Veh, FUS‐tg‐Ril, and FUS‐tg‐Cel groups, but not in FUS‐tg‐NC animals, in comparison with controls (*F*_2,15_ = 4.6, *P* = .012; *F*_2,7_ = 18.39, *P* = .0068; *F*_2,8_ = 9.27, *P* < .005; *F*_2,19_ = 1.06, *P* = .599; respectively, Tukey's test, Figure 2B). FUS‐tg‐NC did not differ significantly from FUS‐tg‐Veh animals in this measure (*F*_2,11_ = 2.59, *P* = .1827). At Week 6, ANOVA showed significant effects of genotype and treatment for body weight (*F*_1,10_ = 27.98, *P* < .0001, and *F*_3,10_ = 3.499, *P* = .0182), but not for their interaction (*F*_3,10_ = 0.7239, *P* = .53499). FUS‐tg‐Veh animals showed a significant decrease in body weight in comparison with WT‐Veh group (*F*_2,15_ = 34.72, *P* = .015, Figure 2C) that was not observed in FUS‐tg‐treated groups (*P* > .05).

ANOVA revealed significant genotype effect for liquid intake (*F*_1,57_ = 11.78, *P* = .0011), but not treatment and a tendency to significance in their interaction (*F*_3,57_ = 0.6534, *P* = .5841 and *F*_3,57_ = 2.404, *P* = .0769). There was significant genotype effect for food intake (*F*_1,71_ = 10.58, *P* = .0018), but not treatment effect, nor their interaction (*F*_3,71_ = 1.192, *P* = .3189, and *F*_3,71_ = 1.136, *P* = .3404). In comparison with WT‐Veh group, both parameters were significantly decreased in FUS‐tg‐Veh mice (food intake: *F*_2,29_ = 10, *P* = .0018, liquid intake: *F*_3,29_ = 11.78, *P* = .0011; Tukey's test; Figure 2D,E), but not in mutants that received either treatment (*P* > .05). Together, in a course of the development of ALS pathology, FUS‐tg‐Veh mice showed a decline in physiological readouts, which was partially counteracted by all applied treatments, and to the greater extend by Neuro‐Cells infusion.

#### 3.1.2 Motor functions

**Figure 3:**
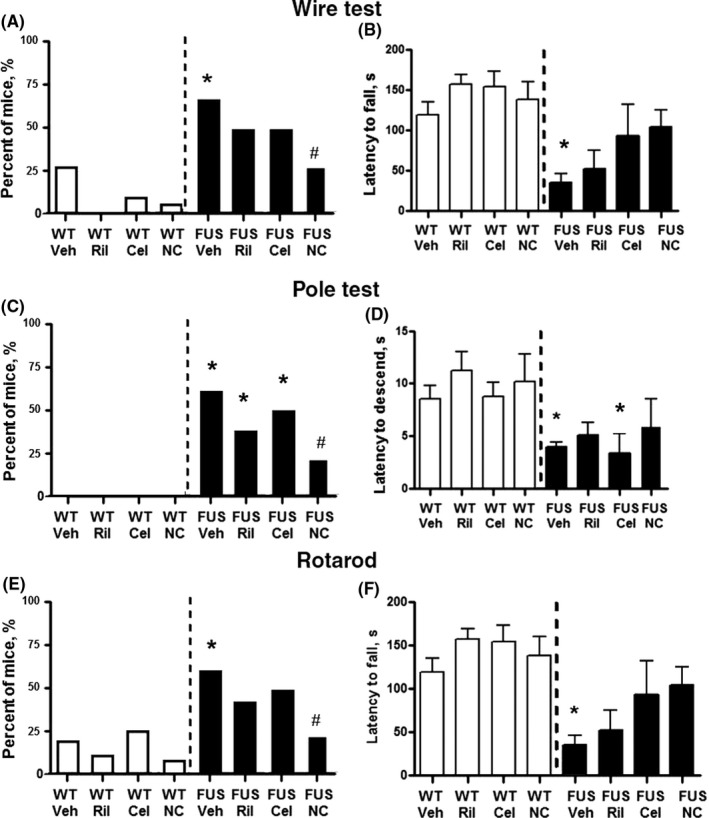
Motor parameters in FUS‐tg mice and effects of riluzole, celecoxib, or Neuro‐Cells. In comparison with WT‐Veh mice, FUS‐tg‐Veh group showed a significant (A) increase in the number of mice with falling events and (B) decrease in the latency to fall in the wire test, that was not found in FUS‐tg animals that received either treatment (*P* > .05). FUS‐tg‐NC mice had a significantly lower number of mice with falling events. In the pole test, in comparison with controls, FUS‐tg‐Veh, FUS‐tg‐Ril, FUS‐tg‐Cel, but not in FUS‐tg‐NC had a significantly (C) higher number of mice with sliding events and (D) decrease in the latency to descend. The latter group had a significantly fewer mice with sliding events than the FUS‐tg‐Veh group. In the rotarod, in comparison with WT‐Veh animals, FUS‐tg‐Veh mice, but not mutant mice that received either treatment, had (E) higher number of mice with falling events and (F) a significant decrease in the latency to fall. FUS‐tg‐NC mice had a significantly fewer number of animals displaying falling in comparison with the FUS‐tg‐Veh group; this group and riluzole‐treated FUS‐tg mice did not show changes in the latency to fall in comparison with the WT‐Veh mice (*P < .05 vs WT‐Veh group, #*P* < .05 vs FUS‐tg‐Veh group; left panels: exact Fisher's test, right panels: two‐way ANOVA and Tukey's test). Each WT‐group was comprised of 10 animals; 7‐15 mice were used in FUS‐tg groups (FUS‐Veh, n = 15; FUS‐Ril, n = 10, FUS‐Cel, n = 7, FUS‐NC, n = 9)

No significant group differences were found in motor tests till sixth week of the study, on weeks 1‐5 (*P* > .05; data not shown). At sixth week, in the wire test, the number of animals that has fallen from the wire was significantly higher in FUS‐tg‐Veh group than in controls (*P* = .018, Fisher's test; Figure 3A), that was not the case for groups of mutants subjected to either treatment (*P* > .05). In addition, this measure was significantly lower in FUS‐tg‐NC mice than in FUS‐tg‐Veh mice (*P* = .0028, Fisher's test). ANOVA showed significant effects of genotype and treatment for the latency to fall (*F*_1,89_ = 24.52, *P* < .0001 and *F*_3,89_ = 2.867, *P* = .0410), but not for their interaction (*F*_3,89_ = 1.126, *P* = .3431) and a significant decrease of this parameter in FUS‐tg‐Veh mice in comparison with WT‐Veh mice (*P* = .011, Tukey's test; Figure 3B). No such differences were observed in FUS‐tg mice that received either treatment (*P* > .05).

In the pole test, in comparison with WT‐Veh animals, the number of animals with sliding events was significantly higher in FUS‐tg‐Veh, FUS‐tg‐Ril, and FUS‐tg‐Cel groups (*P* = .0023, *P* = .018, and *P* = .001, respectively; Fisher's test; Figure 3C), but not in the FUS‐tg‐NC group (*P* = .38). This measure was significantly smaller in the latter group than in FUS‐tg‐Veh group (*P* = .0012; Figure 3D). There were significant showed significant effects of genotype for the latency to descend (*F*_1,77_ = 29.03, *P* < .0001, two‐way ANOVA), but not for treatment and their interaction (*F*_3,77_ = 1.471, *P* = .2289, and *F*_3,77_ = 0.1812, *P* = .9089). This measure was significantly shorter in FUS‐tg‐Veh and FUS‐tg‐Cel animals than in controls (*P* = .025 and *P* = .032¸ respectively; Tukey's test; Figure 3F) suggesting sliding of these mice and motor deficits. No such changes were shown for FUS‐tg‐Ril and FUS‐tg‐NC animals (*P* = .32 and *P* = .55, respectively).

The number of animals falling off the rotarod test was significantly higher in FUS‐tg‐Veh group than in WT‐Veh controls (*P* = .029, Fisher's test; Figure 3E). No such differences were observed in mutants from the treatment groups (*P* > .05); this parameter was significantly lower in FUS‐tg‐NC mice than in FUS‐tg‐Veh group (*P* = .0015). ANOVA showed significant effects of genotype for the latency to fall in this test (*F*_1,99_ = 14.36, *P* = .0003), but not for treatment and their interaction (*F*_3,99_ = 1.431, *P* = .2383, and *F*_3,99_ = 1.048, *P* = .3747). A significant decrease in this measure was found in FUS‐tg Veh group as compared to WT‐Veh group (*F*_3,29_ = 7.28, *P* = .023 Tukey's test; Figure 3F), but not in the groups mutants that received either treatment (*P* > .05). Thus, all treatments prevented the occurrence of significant motor decline in FUS‐tg mice in comparison with controls, whereas only the Neuro‐Cells infusion resulted in a significant improvement in motor performance in comparison with vehicle‐treated mutants. While housing on celecoxib‐containing food pellets resulted in a nonsignificant decrease of body weight in a control group, this factor has unlikely interfered with the evaluation of ALS syndrome in celecoxib‐treated mutants, since no differences in behavioral and physiological parameters were found between naïve and celecoxib‐treated controls.

### 3.2 Histological hallmarks of ALS syndrome in FUS‐tg mice and effects of Neuro‐Cells

**Figure 4:**
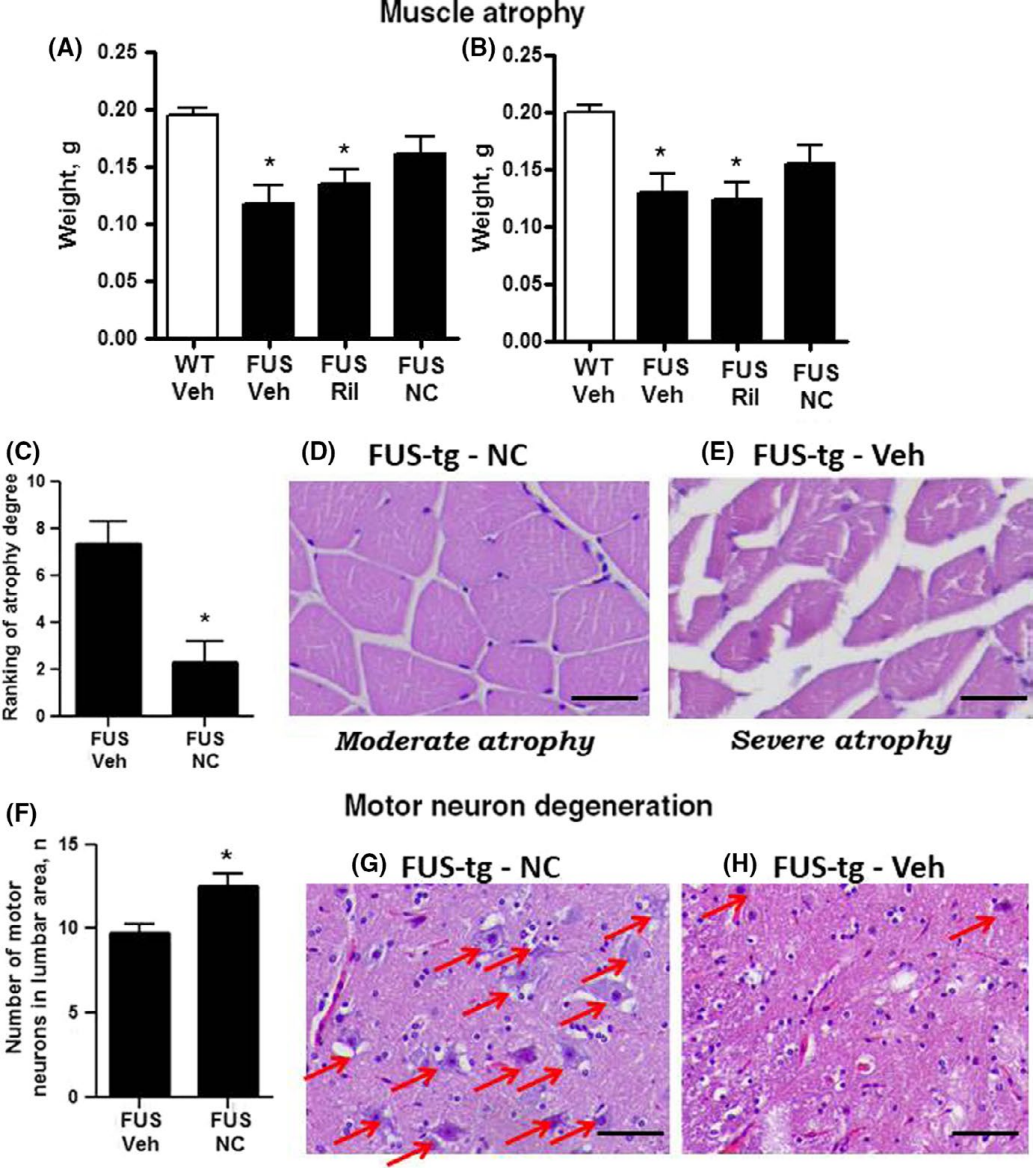
Histological hallmarks of ALS‐like syndrome in FUS‐tg mice treated with Neuro‐Cells. In comparison with WT‐Veh, FUS‐tg‐Veh, FUS‐tg‐Veh and FUS‐tg‐Ril showed a significant decrease in the mass of muscles gastrocnemius of (A) left and (B) right limbs (*P < .05 vs WT‐Veh group) that was not observed for FUS‐tg‐NC group (*P* > .05; 6‐10 mice were used: WT‐Veh, n = 10, FUS‐Veh, n = 10; FUS‐tg‐Ril, n = 8, FUS‐tg‐NC, n = 6). C, A score of atrophy between (D) moderate and (E) severe cases (extreme histological examples in mice from FUS‐tg‐NC (n = 5) and FUS‐tg‐Veh (n = 6) groups) revealed significantly lower scores for FUS‐tg‐NC than FUS‐tg‐Veh mice (P* < .05, Mann‐Whitney). F, The number of motor neurons was significantly higher in FUS‐tg‐NC of FUS‐tg‐Veh mice (P* < .05, Mann‐Whitney, 6 mice per group were used). H,G, Photomicrographs exemplifying the range of motor neuron number in the ventral horn of the lumbar cord in FUS‐tg‐NC of FUS‐tg‐Veh mice in lumbar parts of the spinal cord, respectively. Scale bar = 20 μm

There was a significant difference group difference in mass of the left and right muscle gastrocnemius (*F*_3,29_ = 11.30, *P* = .004, and *F*_3,29_ = 12.35, *P* < .001, respectively, one‐way ANOVA). In comparison with WT‐Veh group, FUS‐tg‐Veh and FUS‐tg‐Ril mice, but not FUS‐tg‐NC animals, showed a significant decrease in these measures (*P* = .004 and *P* = .008, *P* = .002 and *P* = .0016, *P* = .273 and *P* = .3165, respectively, Tukey's test; Figure 4A,B). Because FUS‐tg‐Ril mice showed a loss of muscle mass similar to that of FUS‐tg‐Veh animals, further assessment of muscle atrophy using a histological assay was carried out FUS‐tg‐NC mice that were compared against FUS‐tg‐Veh animals. Histological analysis revealed significant atrophy of muscle gastrocnemius in FUS‐tg‐Veh mice (Figure 4C‐E). FUS‐tg‐NC group had significantly lower scores of muscle degeneration than FUS‐tg‐Veh mice (*U* = 9.6, *P* = .037, Mann‐Whitney). Thus, muscle atrophy in mutants was partly rescued by Neuro‐Cells infusion.

Next, we examined the effects of Neuro‐Cells administration on the motor neuron degeneration at a time corresponding to the onset of the ALS syndrome and the muscle atrophy, as mentioned above. At this time, there was a significant loss (40%‐60%) of motor neurons in the FUS‐tg mutants.^9^ FUS‐tg‐NC group displayed improved motor scores in comparison with other groups as well as higher muscle mass as compared to Ril‐treated mutants. Thus, motor neuron counts in the lumbar L3‐L5 mouse spinal cord were studied in FUS‐tg mice treated with either vehicle or Neuro‐Cells. We found that the number of motor neurons in the lumbar parts of the spinal cord of FUS‐tg‐NC animals was significantly higher than that of FUS‐tg‐Veh animals (*U* = 2.62, *P* = .039, Figure 4F‐H).

### 3.3 Pro‐inflammatory changes and altered activities of GSK‐3 in FUS‐tg mice: effects of celecoxib and Neuro‐Cells

**Figure 5:**
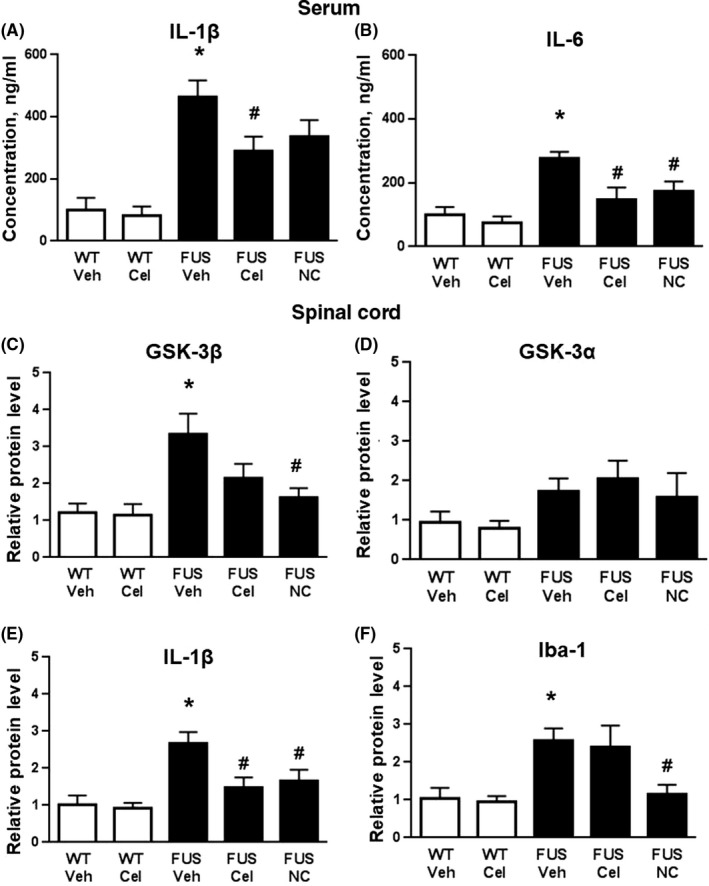
Effects of celecoxib and Neuro‐Cells on the expression of molecular markers of inflammation and GSK‐3 in FUS‐tg mice. A, In comparison with controls, animals from FUS‐tg‐Veh and FUS‐tg‐NC groups displayed increased serum concentrations of IL‐1β; FUS‐tg‐Cel group displayed reduced values of this measure in comparison with FUS‐tg‐Veh mice. B, In comparison with controls, FUS‐tg‐Veh, but not FUS‐tg‐Cel and FUS‐tg‐NC, had elevated serum IL‐6. Two later groups had significantly lower serum IL‐6 concentrations than FUS‐tg‐Veh group. In comparison with WT‐Veh mice, there were significantly elevated protein levels of (C) GSK‐3β, (E) IL‐1β and (F) Iba‐1 in spinal cords of FUS‐tg‐Veh animals; but not in FUS‐tg‐Cel and FUS‐tg‐NC groups. D, No significant differences were found in the protein levels of GSK‐3α (*P* > .05). FUS‐tg‐NC group showed significantly lower values of protein expression of GSK‐3β, IL‐1β, and Iba‐1 than FUS‐tg‐Veh mice. FUS‐tg‐Cel group showed a significant decrease of IL‐1β expression in comparison to FUS‐Veh group. (*P < .05 vs WT‐Veh group, #*P* < .05 vs FUS‐tg‐Veh group: one‐way ANOVA and Tukey's test). 5‐9 mice per group were used: 6 mice in WT‐Veh group, 5 mice in WT‐Cel and FUS‐Cel groups, and 9 mice in FUS‐Veh and FUS‐NC groups

There were significant group differences in serum concentrations of IL‐1β and IL‐6 (*F* = 10.62 *P* < .001, and *F* = 11.74, *P* < .001, one‐way ANOVA, respectively). In comparison with WT‐Veh mice, these measures were significantly elevated in FUS‐tg‐Veh group (*P* = .012 and *P* = .035, respectively; Tukey's test; Figure 5A,B). No such increases in IL‐6 were found for FUS‐tg‐Cel and FUS‐tg‐NC groups (*P* = .29 and *P* = .22, respectively). These groups showed a significant decrease in this measure in comparison with the FUS‐tg‐Veh mice (*P* = .01 and *P* = .025, respectively). In comparison with FUS‐tg‐Veh group, serum levels of IL‐1β were significantly lower in FUS‐tg‐Cel mice and nonsignificantly lower in FUS‐tg‐NC mice (*P* = .042 and *P* = .065, respectively; Figure 5A); no other significant differences were found.

Significant group differences in protein levels were found for GSK‐3β, IL‐1β, and Iba‐1 (*F* = 4.992, *P* = .0035; *F* = 6.784, *P* = .0006; and *F* = 5.605, *P* = .0018) but not GSK‐3α (*F* = 1.359, *P* = .272). In comparison with WT‐Veh mice, expression of GSK‐3β, IL‐1β, and Iba‐1, but not GSK‐3α was significantly elevated in FUS‐tg‐Veh animals (*P* = .026, *P* = .013, *P* = .041, and *P* = .14, respectively, Tukey's test; Figure 5C‐F; for original Western blot images used for quantification see Figure S6). All increased parameters were significantly lower in FUS‐tg‐NC mice than in the latter group (*P* = .0027, *P* = .011, and *P* = .036, respectively); similar decreases were found in FUS‐tg‐Cel group for IL‐1β, but not for GSK‐3β and Iba‐1 (*P* = .017, *P* = .181, and *P* = .785, respectively). Thus, both administration of celecoxib and Neuro‐Cells decreased, but only the latter treatment has significantly lowered expression of markers of microglial activation Iba‐1 and GSK3β in the spinal cord.

### 3.4 Effects of administration of Neuro‐Cells infusion in SOD‐1 mice

**Figure 6:**
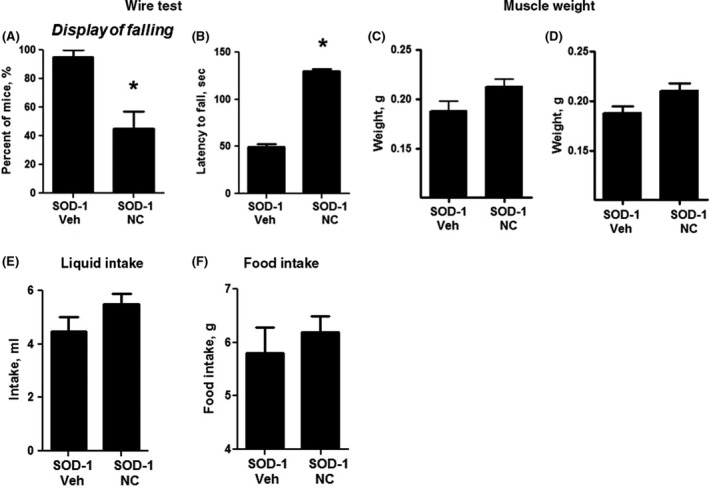
Effects of Neuro‐Cells infusion in SOD‐1 mice. A, There were significantly fewer numbers of mice displaying the falling in the wire test in SOD‐1‐NC mice than in SOD‐1‐Veh mutants. B, The latency to fall in the wire test was significantly longer in SOD‐1‐NC mice than in SOD‐1‐Veh. C, D, There was nonsignificant trend to higher muscle weight in SOD‐1‐NC than in SOD‐1‐Veh group for left and right muscles gastrocnemius, respectively, and for (E,F) higher liquid and food intake in SOD‐1‐NC mice than in SOD‐1‐Veh animals (*P < .05 vs SOD‐1‐Veh group). 5 mice were used in SOD‐1‐Veh group, and 7 mice were used in SOD‐1‐NC group

There were no significant differences in body weight between SOD‐1‐Veh and SOD‐1‐NC groups during weeks 1‐9 (*P* > .05; Mann‐Whitney test). The number of mice fallen from the wire was significantly lower, and the latency to fall was significantly longer in SOD‐1‐NC than in SOD‐1‐Veh groups (*P* = .028, Fisher test; and *U* = 4.5, *P* = .003; Figure 6A,B). No other group differences were found (*data not shown*). There was a trend to higher muscle weight in SOD‐1‐NC animals in comparison with SOD‐1‐Veh mice for left and right muscles gastrocnemius (*U* = 6.5, *P* = .081, and *U* = 7, *P* = .10, respectively; Figure 6C,D) and an optical trend to higher liquid and food intake in SOD‐1‐NC mice (*U* = 7, *P* = .10, *U* = 3.6, *P* = .16; Figure 6E,F). Thus, Neuro‐Cells infusion to SOD‐1 mutants resulted in overall improved functional readouts in these mice.

## 4 DISCUSSION

Our study supports the hypothesis that the new stem cell preparation Neuro‐Cells ameliorates an experimental ALS syndrome. A single i.c.v. infusion of Neuro‐Cells into FUS‐tg mutants counteracted the development of neurodegenerative changes, muscle atrophy, motor deficits, and general physical decline and was associated with a decrease in inflammatory markers. These findings are in accordance with recently published results demonstrating beneficial functional and anti‐inflammatory action of Neuro‐Cells in a rat model of spinal cord injury.^23^ The effects of Neuro‐Cells in FUS‐tg mice were compared against standard therapies with riluzole and classic anti‐inflammatory drug celecoxib. These standard treatments partially ameliorated ALS pathology, but these improvements were less marked than the effects of Neuro‐Cells. The administration of Neuro‐Cells, but not celecoxib, normalized the expression of Iba‐1, a marker of microglial activation and GSK‐3ß expression in the spinal cord. While the administration of celecoxib altered cytokine expression in the mutants in a similar manner as Neuro‐Cells, it did not alter the expression of Iba‐1 and GSK‐3ß in the mutants.

The findings concerning Iba‐1 and GSK‐3ß expression support the view that there is a critical role for microglial activation and inflammation in ALS.^14^, ^15^, ^16^, ^17^, ^18^ The upregulation of GSK‐3ß cascade is known to be functionally associated with pro‐inflammatory changes and microglial activation, which is a well‐established marker of ALS histopathology, and the FUS‐ALS forms in particular.^59^, ^60^ GSK‐3ß activities are inversely related to neurotrophin expression, and its downregulation is another recognized molecular feature of human ALS.^15^, ^18^, ^61^ GSK‐3ß is also implicated in the mechanisms of microglial activation during neurodegeneration.^62^ Thus, present study suggests that microglial activation and increased GSK‐3ß activities could be targets for ALS therapy, as they were normalized by the administration of the stem cell therapy.

It is a generally held view that the anti‐inflammatory effects of stem cells are considered to be attributable to the paracrine activity of anti‐inflammatory cytokines and growth factors, as well as the release of extracellular vesicles containing anti‐inflammatory and anti‐apoptotic proteins.^22^, ^63^, ^64^ These processes are likely to act on RNA‐dependent and RNA‐independent elements of FUS‐related pathology.^6^, ^11^, ^65^, ^66^ Transplanted bone marrow derived stem cells were shown to secrete such anti‐inflammatory cytokines and growth factors, as IL‐10, vascular endothelial growth factor (VEGF), insulin‐like growth factor‐1 (IGF‐1), hepatocyte growth factor (HGF), brain‐derived neurotrophic factor (BDNF), neuronal growth factor (NGF), and others.^67^, ^68^ This secretion was shown to be condition‐dependent, and in particular, the presence of inflammation was found to stimulate the release of the anti‐inflammatory molecules.^67^ Given that FUS protein is considered to be functionally related with insulin/IGF‐signaling pathway,^69^ an important element of which is BDNF, a ligand of TrkB receptor,^70^ which regulates the mechanisms of cellular stress,^71^, ^72^ it can be suggested that paracrine secretion of Neuro‐Cells cytokines and growth factors may underlie the anti‐inflammatory and antidegenerative changes observed in the FUS‐tg mice.

In addition, FUS protein can regulate the serine‐threonine protein kinase (Ak strain transforming, Akt) and forkhead box O1 (FOXO) proteins,^58^ further elements of GSK‐3β cascade that can explain the reported changes in GSK‐3β expression in the FUS‐tg model. Moreover, paracrine secretion by stem cells of trophic factors can ameliorate decreased vascular network density in lumbar spinal cords, a recently demonstrated disease mechanism in employed here FUS‐tg mice, which is sensitive to a therapy with angiogenetic/growth factors.^73^ Thus, it might be speculated that the effects of the stem cells might be due to paracrine activities and the release of neurotrophins and anti‐inflammatory cytokines that classical drug therapy cannot provide.^22^

We found that the physiological parameters and performance of FUS‐tg mice treated with Neuro‐Cells compared to mutants receiving other therapies were better preserved. In particular, celecoxib‐treated mutant mice exhibited limited amelioration of the functional endpoints and no changes in the Iba‐1 or GSK‐3ß expression. These data may go some way to explain the reported failures of using anti‐inflammatory drugs during ALS.^19^, ^20^, ^21^ Nevertheless, standard ALS and anti‐inflammatory treatment induced partial ameliorative effects on the ALS‐related abnormalities that validates the FUS‐tg line as a model of ALS.

To verify results obtained on FUS‐tg mice, we performed the i.c.v. infusion of Neuro‐Cells in SOD‐1 mutants, the classic ALS model. The results indicated that there were improved motor functions under these conditions following Neuro‐Cells administration. Together, our data provide evidence of the disease‐counteracting and anti‐inflammatory effects of Neuro‐Cells in two experimental models of ALS and are in line with previously published reports on effectiveness of stem cell therapy during this disease,^37^, ^74^ as well as with previous findings with this preparation applied in a spinal cord injury model.^23^ They also highlight the importance of compliance in preclinical ALS studies with the internationally accepted guidelines 24 to more accurately approximate the usefulness of novel therapeutics (see Data S1), including stem cells and thus provide a better basis for clinical trials.

## 5 CONCLUSIONS

Our study provides the first evidence for greater efficacy of Neuro‐Cells as a therapeutic option capable of counteracting the progression of ALS‐like pathology and accompanying inflammatory changes in comparison with the standard pharmacological references. As discussed above, it is very likely that both MSCs and HSCs types of cells contribute to the effect seen in the study. While beneficial effects of HSCs are likely to be due to their well characterized roles in neuropoiesis and production of trophic factors, MSCs, besides their crucial function in the regulation of HSCs functions, might ameliorate angiogenesis and vascular density and exert anti‐inflammatory effects. Further research is needed to define the mechanisms of anti‐inflammatory action and other effects of Neuro‐Cells on hallmarks of ALS syndrome. Yet, at this stage, stem cell therapy should be considered as part of therapeutic approach to the treatment of ALS.

## CONFLICT OF INTEREST

HdM and EW are CEOs of Neuroplast BV, Maastricht, Netherlands, and have filed a patent describing the Neuro‐Cells preparation that was used as one of three treatments in this study. The application number of the patent filing is 15/525328. TS is a consultant at Neuroplast BV.
