# Supplementary material for: Neuro‐Cells therapy improves motor outcomes and suppresses inflammation during experimental syndrome of amyotrophic lateral sclerosis in mice
Source: CNS Neurosci Ther. 2019 Dec 23;26(5):504–17. doi: 10.1111/cns.13280 (PMC7163689; doi:10.1111/cns.13280)
Supplement: Supplementary file 1 [file CNS-26-504-s001.docx]

**Supplementary File**

**Animals and housing**

Mouse colony of FUS-tg mice and their wild type littermates (WT) was bred in the FDA-certified SPF facilities of IPAC; generation of FUS-tg mice is described below. SOD-1 mutant mice were obtained from Charles River provider (see main text). Starting from two weeks prior to the experiment, six-weeks-old male mice were single-housed under standard conditions (12 hrs light/dark cycle, lights on at 21:00, humidity 50–60%, temperature 22±1°C) with food and water *ad libitum*. All efforts were undertaken to minimize the potential discomfort of experimental animals.

## Generation of FUS-tg mice

Generation of FUS-tg mice was performed as described elsewhere (Schelkovnikova et al., 2013). Briefly, a fragment of human FUS [1–359] cDNA including 9 bp of 5′-UTR was cloned into Thy-1 promoter plasmid 323-pTSC21k. A gel-purified fragment obtained by digestion of the resulting plasmid DNA with NotI was used for microinjection of mouse oocytes. Transgenic animals were identified by PCR analysis of DNA from ear biopsies by the presence of 255-bp product (primers 5′-TCTTTGTGCAAGGCCTGGGT-3′ and 5′-AGAAGCAAGACCTCTGCAGAG-3′). The original transgenic line on C57Bl6/CBA genetic background was backcrossed with CD1 wild type mice for several (>7) generations.

**Characteristics of human marrow stem cells**

“Neuro-Cells” is a standardized preparation of fresh, unmanipulated human bone marrow-derived stem cells, which is produced under regulations of good manufacturing practices (GMPs) without expansion and/or labeling (de Munter et al., 2019; patent WO2015/059300A1). Neuro-Cells comprise mesenchymal stem cells and their progenitors (MSCs), hematopoietic stem cells and their progenitors (HSCs), and other mononuclear cells (*Table 1*).

**Table 1. Cell populations in ''Neuro-Cells" preparation**

| **Characterization of cell populations in ''Neuro-Cells" preparation** | |
| --- | --- |
| **Cell populations in Neuro-Cells** | **Absolute number of cells injected per mouse in 10**  ul |
| Total number of nucleated cells | 1.39 x 10^6^/10 ul |
|  |  |
| **HSC**: total CD34^+^ cells (calculated by FACs) | 5.00 x 10^5^/10 ul |
| Other mononucleated cells | 8.9 x 10^5^/10 ul |
| The proportion of MSCs expressing CD markers in overlapping manner (in total cell preparation, defined by single staining assay): |  |
| **MSC**: CD271^+^ cells (7%) |  |
| **MSC**: CD90^+^ cells (13%) |  |
| **MSC**: CD105^+^ cells (85.9%) |  |
| **MSC**: CD73^+^ cells (4%) |  |

**Production of human marrow stem cells**

For production of Neuro-Cells, healthy volunteer donors with informed consent were recruited for collection of 50-75 mL of bone marrow from their iliac crest under local anesthesia following standard operating procedures. Briefly, mononuclear cells were isolated by Ficoll density gradient centrifugation (1.077 g/ml; Sigma, Munich, Germany) at 400×g for 35 minutes. The mononuclear cells were re-suspended in the culture medium composed of DMEM (GIBCO, Rockville, MD, USA) with low glucose concentration and 10% fetal bovine serum (FBS; Hyclone, Logan, UT, USA). The mononuclear cells were plated at 1×10^6^ cells/25 cm^2^ in culture flasks and the cultures were incubated at 37°C in 5% CO_2_ in the air and 95% humidity. The medium was exchanged after 48 hours and then every 3 to 4 days. When the cultures reached approximately 90% of confluence, human marrow stem cells (“Neuro-Cells”) were passaged with 0.25% trypsin (GIBCO) and re-plated into passage culture at a density of 5000 to 10,000 cells/cm^2^. Cells at passages 3 and 4 were used for transplantation. Upon harvest, the cells were isolated by treatment with 0.25% trypsin.

The cells were then washed four times and re-suspended in PBS at a density of 100,000 cells/μl. Viability of the cells was assessed using a 0.4% Trypan Blue dye (Sigma, Berlin, Germany) exclusion method prior to and following transplantation. The cells were analyzed for their immunophenotype by flow cytometry (Beckman Coulter, Fullerton, CA, USA). Fluorescein isothiocyanate (FITC)-conjugated or phycoerythrin-conjugated antibodies specific for human CD14, CD19, CD45, CD34, CD73, CD90, CD105, and HLA-DR tested for flow cytometry application. The percentage of positive cells was determined based on fluorescence emission of the nonspecific FITC/phycoerythrin isotypic antibody controls.

A preparation of Neuro-Cells was aliquoted, deeply frozen and stored at -80 C° until the use. A standard thawing procedure was applied on the day of the administration of Neuro-Cells (*see below*). Cells were re-suspended in Ringer Lactate buffer, their vitality was evaluated as described above and concentration in buffer was adjusted to 500,000 CD34^+^ in 10 µl for i.c.v. injections; vials were kept in +4C°. *Table 1* presents an overview of the number of cells injected per a mouse.

**Thawing of Neuro-Cells preparations**

For all experiments with i.c.v. administration of Neuro-Cells, preparations from the same stock sample were arranged *ex temporo*. The standard thawing procedure is described in *Table 2*.

**Table 2: Standard thawing procedure for Neuro-Cells**

| **Nr.** | | **Step 1** | **Set** |
| --- | --- | --- | --- |
| 1 |  |  |  |
|  |  | sterile Ringer Lactate solution is used  pre-warm at 37°C water bath | 50ml  pre-warmed 37°C |

| **Nr.** | | **Step 2** | **Set** |
| --- | --- | --- | --- |
| 1 |  | To thaw frozen Neuro-Cells, cryovials are placed in a water bath at 37°C until only a small sliver of ice is present; caution is paid to prevent cryovial cap from submerging in the water. | 37°C |
| 2 |  |  |  |
|  | A | - Cyovials are transferred to the laminar flow hood  - all vials are wiped with 70% IPA before opening the cap using a 70% IPA soaked tissue |  |
|  | B | each thawed cell type is transferred into an individual 15ml tube containing 5ml pre-warmed thawing buffer | 5ml |
|  | C | - cryovials are rinsed with fresh pre-warmed thawing buffer  - pooled cells are mixed by gently pipetting up/down twice | Rinse 2x |
|  | D | Suspension is centrifuged under 300g for 5 min | 300g 5 min |
|  | E | - supernatant is completely aspirated  - the cell pellet are handled carefully  - pellet is resuspended into the **thawing buffer for washing** |  |
|  | F | For washing, pellet is centrifuged under 300g for 5 min | 300g 5 min |
|  | G | supernatant is completely aspirated  -- pellet is resuspended into the **thawing buffer for washing**  **- cells are pooled where applicable** |  |

**Vitality assessment of Neuro-Cells preparations after thawing**

After thawing, сell counting was performed using the "Countess II FL Automated Cell Counter" (Thermo Fisher Scientific AMQAF1000, Toronto, ON, Canada) according to the manufacturer's protocol. Three batches of cells from the same bone marrow extraction were used in the study.

**Set 1**

**Set 2**

**Set 3**

We checked viability of Neuro-Cells samples used in the study and ensured high vitality percentages in samples, that were ranging from 62 to 68% Subsequent flow cytometry was done and HSC numbers were adjusted for injection.

**Pilot study to define conditions of i.c.v. infusion of Neuro-Cells with stereotaxic surgery**

A pilot study was conducted to define optimal conditions of the i.c.v. infusion of Neuro-Cells to mice with stereotaxic surgery. Following conditions of Neuro-Cell administration were tested in a pilot study on 12 CD1 mice: (**1**) 50, 000 cells for 5 min in 10 ul (n=1), (**2**) 50, 000 cells for 10 min in 10 ul (n=1), (**3**) 150, 000 cells for 10 min in 10 ul (n=1), (**4**) 250, 000 cells for 10 min in 10 ul (n=2), (**5**) 500, 000 cells for 10 min in 10 ul (n=2), (**6**) Ringer Lactate buffer, 10 ul (n=2), (**7**) no injection (n=1). (**8**) Additionally, the administration of 10, 000 stem cells obtained from bone marrow of a mutant mouse expressing green fluorescent protein (GFP) (Kovinva et al., 2019) for 10 min in 10 ul (n=2) was carried out in order to compare a diffusion of human stem cell preparation against mouse stem cell preparation. Mice were killed 24 h after injection using halothane. Brain, liver, lungs, spleen were collected and stained for anti-mitochondrial human antigen from experimental sets 1-7, as previously described (Donders et al., 2015).

Briefly, mice were deeply anesthetized by isoflurane inhalation and perfused transcardially with Ringer lactate (pH 7.4) and 4%-parapharmaldehyde (Sigma-Aldrich) in 30% PBS-sucrose (Merck Chemicals N.V., Overijse, Belgium). Brains, lungs, lymph nodes, spleen were dissected, positioned into fixative solution for 24 h. Subsequently, tissues were snap frozen in the gas phase of liquid nitrogen and stored at -80°C until further processing. Cryosections (10 µm) were cut and stored at -20°C until further processing. Staining for human mitochondria, a marker for cells of human origin in mouse tissue, was performed. Tissue slices were obtained with a Leica CM3050 S cryostat (Leica, Groot-Bijgaarden, Belgium). The protocol of immunostaining with anti-human mitochondria was previously validated to detect injected cells in a rat study using double staining for C68-positive cells (Donders et al., 2015). Slides were washed with Tris-buffered saline (VWR) containing 0.5% Tween-20 (Merck Chemicals) (TBS-T), blocked with 10% normal goat serum (Dako, Heverlee, Belgium) in TBS-T for 45 min and subsequently incubated overnight with mouse anti-human mitochondria antibody (1:800 in TBS-T; clone 113-1; Millipore, Merck Chemicals, Frankfurt, Germany). Control stainings were performed by omitting the primary antibodies. Brain tissue was counterstained with 0.1% cresyl violet. Lungs and spleen were counter stained with haematoxylin. Sections were dehydrated and sealed with Entellan mounting medium (Millipore) and imaged and immediately analyzed using a Nikon Eclipse 80i microscope and processed with NIS Elements BR 4.0 software (Nikon Instruments BeLux, Brussels, Belgium).

Material from mice treated with GFP-containing stem cells (set 8) was visualized using a fluorescent microscope. Results from these assays are presented *below*.

**Western Blot**

Tissue samples were treated with lysis buffer containing 20 mM of Tris-HCl (pH 7.5), 450 mM of NaCl, 1%-solution of Triton X-100, 1 mM of EDTA, 1 mM of NaF, 1 mM of Na_3_VO_4,_ and protease inhibitor (Roche Diagnostics, Indianapolis, IN, USA); 50 μl of buffer per 1 g of tissue was used. Samples were then centrifuged at 16 000 rpm for 20 min at 4°C; supernatant was collected and stored until use at -20°C. 25 μg of protein from each sample was mixed with 35 μl of Laemmli buffer. A sample of identical volume, comprising of 26 μl of Laemmli buffer, 5 μl of Page Ruler, and 4 μl of Magic Mark (Sigma, Munich, Germany) was used as a reference. For electrophoresis, samples were diluted in a solution containing MiliQ H_2_O, 1.5 M of Tris Buffer (pH 8.8), 30%-solution of Acrylamide, 10%-solution of SDS Temed, and 10%-solution of ammonium persulfate (APS). For the next step, a solution containing MiliQ H_2_O, 0.5 M of Tris Buffer (pH 8.8), 30%-solution of Acrylamide, 10%-solution of SDS Temed, 10%-solution of APS and gel (Sigma, Munich, Germany) was used. The percentage of gel-containing solution was adjusted to the weight of the protein of interest and was 20% for proteins of the size of 4–40 kDa, 12.5% for proteins of the size of 40–70 kDa, 10% for proteins of the size of 70–100, and 7.5% for proteins over 100 kDa. A buffer containing 25 mM of Tris Base buffer, 192 mM of Glycine (Sigma, Mannheim, Germany), 10%-solution of SDS and MiliQ H_2_O (pH 8.3) was used for gel electrophoresis which was carried out under the constant voltages of 80 V and 130 V.

Polyvinylidene difluoride (PVDF) membrane (9 x 6 cm, EMD Millipore, Billerica, MA, USA) was consequently incubated in a 99%-methanol solution for 1 min (Brocacef, Amsterdam, the Netherlands), a MiliQ H_2_O for 5 min, and a transfer buffer for 15 min. The latter contained 25 mM of Tris Base, 192 mM of glycine, 20%-solution of methanol, and MiliQ H_2_O (pH 8.3). For the next step, blot “transfer sandwich” was composed of buffer-soaked sponge, consisting of two buffer-soaked Whatman filter papers, gel, activated membrane, and ice-cold transfer buffer; a constant current of 300 mA was used for 2 h 30 min.

The membrane was treated with 5%-dry milk solution of TBST, containing 50 mM Tris-HCl (pH=8.2), 150 mM NaCl, 0.05%-solution Tween 20 (Sigma, Munich, Germany) for 1 h at the room temperature and subsequently incubated with primary antibodies (*Table 3*) at 4°C overnight, followed by incubation with respective horseradish peroxidase-conjugated secondary (HRP) antibodies (Sigma-Aldrich, St. Louis, MO, USA) for 2 h at the room temperature on a roller. The membrane was washed in TBST three times, 5 min each time, and then placed on the plastic cover. Then, Western Bright^TM^ ECL kit (Advansta Inc, Menlo Park, CA, USA) was used. Relative optical density of immunoreactive protein bands was examined using ImageJ software (NIH, Bethesda, MD, USA). Results were normalized to the relative intensity of the β-tubulin band that was selected as a reference protein as described elsewhere (Gorlova et al., 2019). Blots were stripped by incubation with Restore Western Blot Stripping Buffer (Thermo Scientific, Rockford, IL, USA) at the room temperature for 15 min.

**Table 3. Primary antibodies used in the Western blot assay**

| **Antibody** | **Dilution** |
| --- | --- |
| Anti-β-tubulin (Abcam, Cambridge, MA, USA) #ab8227 | 1:700 |
| Anti-GSK-3β (Cell Signaling Technology, Beverly, MA, USA) #9832 | 1:800 |
| Anti-GSK-3α (Cell Signaling Technology, Beverly, MA, USA) #4337 | 1:900 |
| Anti-IL-1β (Cell Signaling Technology, Beverly, MA, USA) #12242 | 1:700 |
| Anti-Iba1 (Abcam, Cambridge, MA, USA) ab5076 | 1:500 |

To normalize the data, the relative expression value of each protein of interest was expressed as a percent of the concentration of β-tubulin (the reference protein). The choice of a reference protein was based on the previous observations where its expression was found to vary moderately across various experimental conditions, as well as the linear representation of the intensity of its signal (Gorlova et al., 2019; Pavlov et al., 2019).

**Determination of protein concentration**

Protein concentration was quantified using the BCA protein assay kit (Pierce, Rockford, IL, USA) as described elsewhere (Gorlova et al., 2019; Pavlov et al., 2019). The working reagent was prepared in accordance with manufacturer instructions. 25µl of each standard or sample preparations were pipetted into a microplate well; 200µl of the working reagent was added to each well and mixed thoroughly on a plate shaker for 30 seconds; assay was run in duplicates. The covered plate was incubated at 37°C for 30 minutes and cooled to room temperature for 10 min. The absorbance was measured at 562 nm in a Biotek Microplate Reader (Biotek Instruments, Winooski, VT, USA). Ascent Software Program (Winooski, VT, USA) coupled to the microplate reader was used to calculate protein values based comparing optical densities with the standard curve The standard curve was generated by plotting the average blank-corrected 562 nm measurements for each BSA standard vs. its concentration in µg/ml.

**Study compliance with general and proof of concept study requirements specific to ALS**

The presented study is in compliance with the requirements for any proof of concept (you sure?) and preclinical studies (Ludolph et al., 2010) including the statement of a genetic background and key read-outs, breeding protocols, animal livestock and gender, number of animals per group/subgroup, treatment efficacy. Pharmaca outline was double blind for all experimentators. To examine the onset of clinical traits we examined weight loss, grip strength, wire hanging and rotorod test. Histology was applied for positive outcomes and included motor neuron counts in groups of 5-6 mice. Methods and statistics are described elsewhere. Subsequently, we compared our results with previously published literature.

A sentence regarding any possible conflict of interests, including the sources of financing and tested treatment is provided. The following prerequisites for the conduct of a preclinical animal study with a therapeutic aim present: 1) Recognition of the validity of the model in terms of the ‘genetic validity’ of hereditary/familial form of the FUS-tg condition, and the importance of genetic background that is supported by extensive literature on the employed mouse model. 2) The use two different mouse models of ALS, FUS-tg and SOD-1 AG 93 mice. 3) Validity of the characterization of FUS-tg model is supported by comprehensiveness of undertaken phenotyping. We used classic tests for motor behaviour in mice and classic histological markers of muscular and neuronal degeneration, accompanied by molecular readouts that are well known signs of the disease in a clinic. 4) Our study is a ‘proof of concept’ study as it investigates the role of pathogenetic mechanisms of neuroinflammation that are therapeutically relevant with the ALS that is clearly different from a ‘preclinical drug testing’ pre-clinical trial. 5) It is clearly stated that the intervention is meant to be ‘disease-modifying’. 6) The outcome was replicated in FUS-tg and SOD-1 mice.

**Figure 1**

**
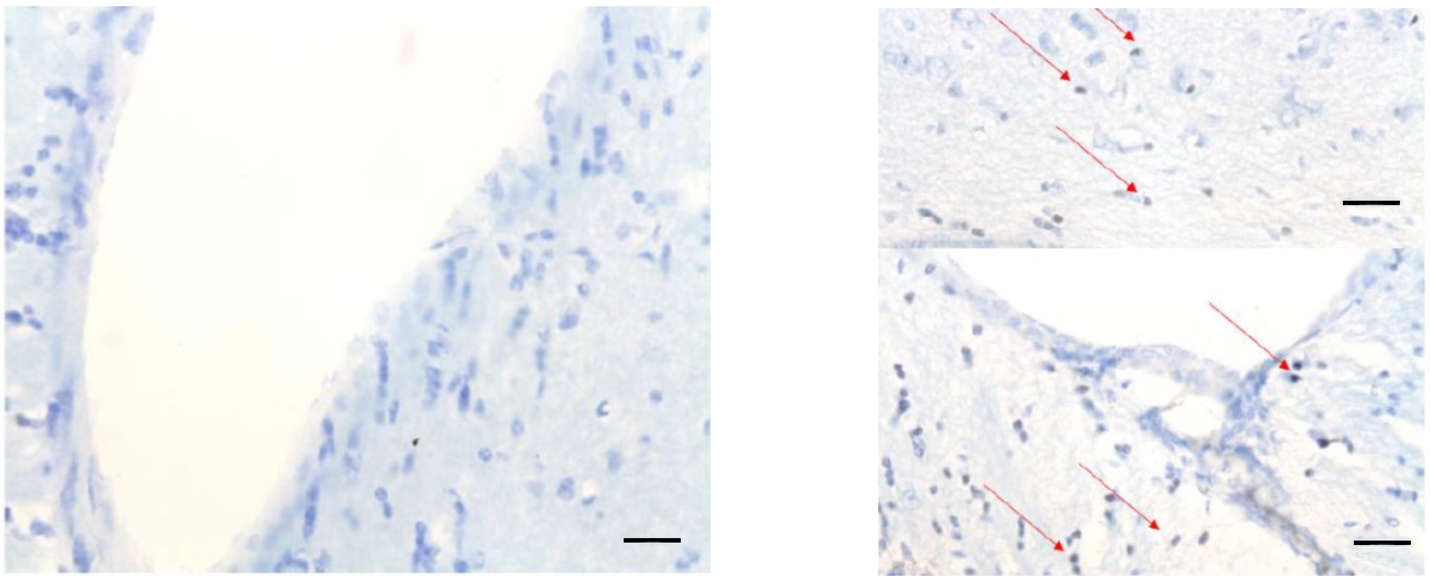
**

***Figure S1.* Identification of i.c.v.-infused Neuro-Cells in the parenchyma surrounding lateral ventricles of the mouse brain.** After 24 hours, mice treated with 250, 000 Neuro-Cells in 10ul of Ringer-lactate buffer could be detected with anti-mitochondrial human antigen within the brain parenchyma of experimental CD1 mice (left: control, vehicle-treated mice, right: Neuro-Cells-injected mice). Anti-human mitochondrial positive staining was observed in regions that were adjacent to the lateral ventricles (red arrows) that were not present in the control. Scale bar = 20um and the tissue was counterstained with cresyl violet.

**Figure 2**

**
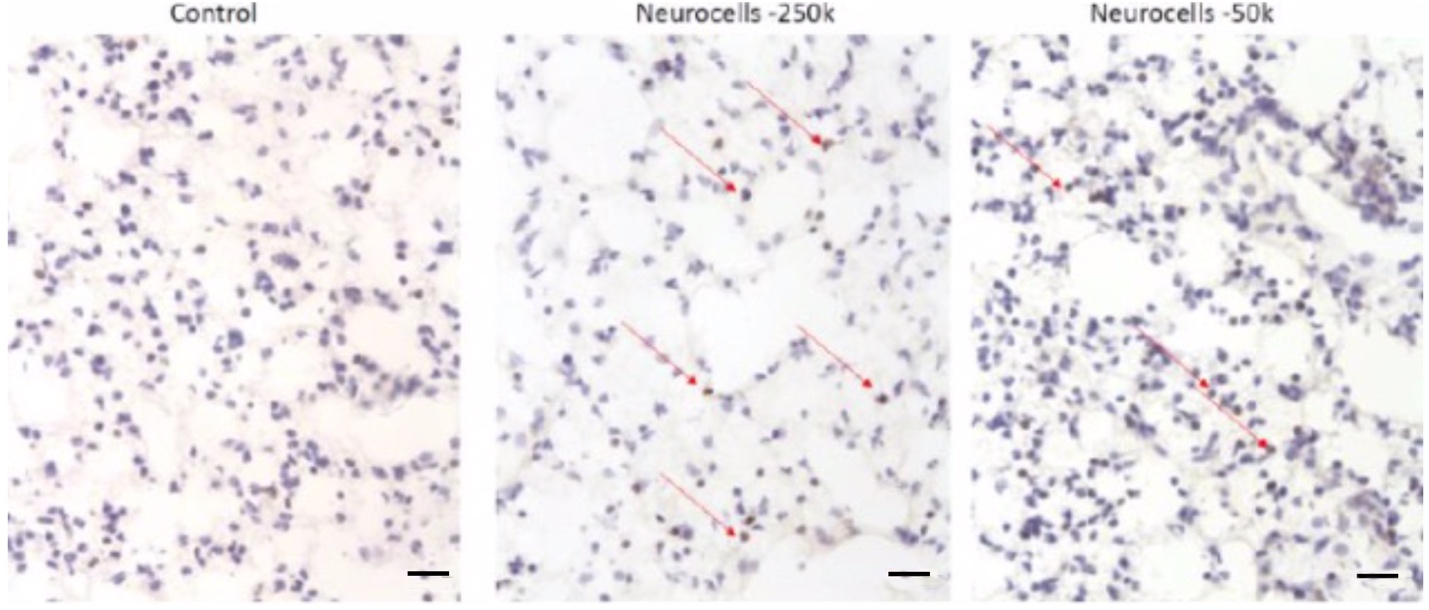
*Figure S2.* Neuro-Cells were detectable in peripheral organs of experimental CD1 mice after the i.c.v.-infusion: anti-human mitochondria staining in the lung.** Mice treated with 50 000 or 250 000 Neuro-cells in 10ul exhibited positive staining for the anti-mitochondrial human antigen in the lung (red arrows), spleen and liver (data not shown). Thus, 24 h post-administration, injected Neuro-cells had managed to leave the brain and enter peripheral organs. It remains unclear how cells injected into the CSF manage to find there way into peripheral organs so rapidly. Scale bar = 20um and the tissue was counter stained with haematoxylin.

**Figure 3**


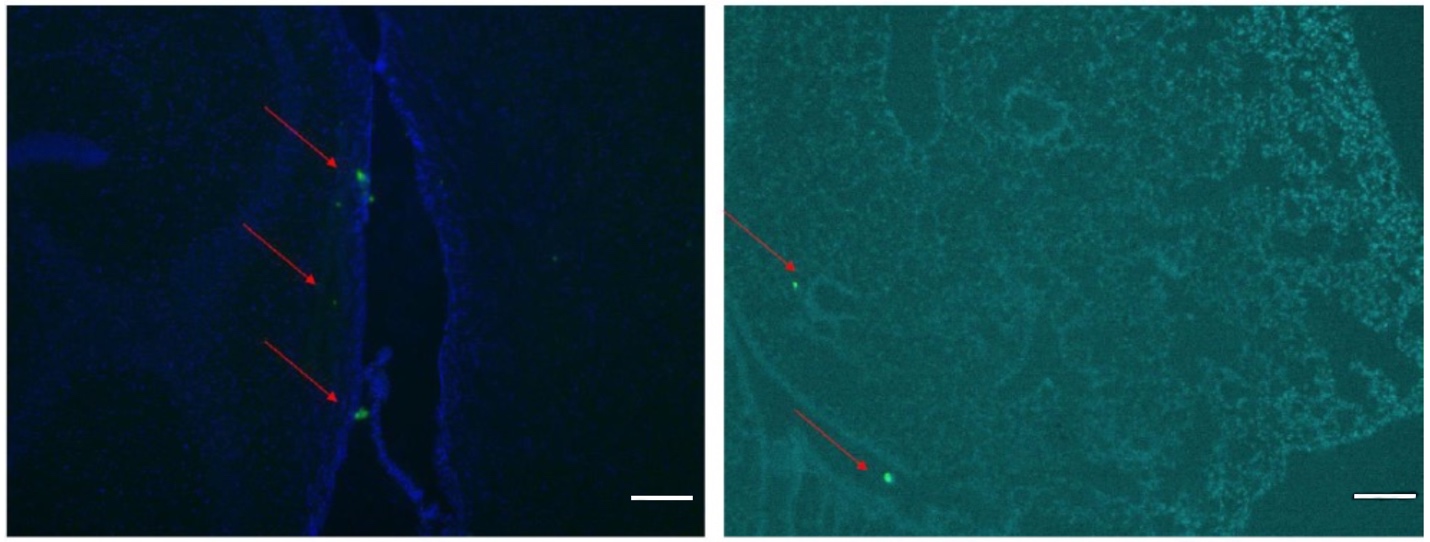

***Figure S3.* Identification of stem cells derived from GFP mice in the brain parenchyma after their i.c.v. infusion to experimental CD1 mice.** To understand how syngeneic stems cells traffic after an i.c.v. injection we injected mouse GFP-expressing bone-marrow-derived stem cells into the CSF. At 24 hours, GFP-positive cells (Kovina et al., 2019) could be detected in the brain parenchyma with a distribution that was similar to that obtained following the administration of Xenogeneic Neuro-Cells. Scale bar = 50um and the tissue was counterstained with DAPI.

**Figure 4**

***Figure S4. Body weight 1 week after surgery and i.c.v. treatment: lack of effects*.** Body weights evaluated after one week in wild type controls and FUs-tg mice that received either i.c.v. administration of Neuro-Cells or vehicle did not differ from body weights of non-operated animals that were housed with tap water (F_2,24_=0.38, p=0.94, two-way ANOVA, post-hoc Tukey’s test). This rules out the possibility of a potential negative impact of surgery on animals’ physical state. Based on this finding, control and FUS-tg mice that received either vehicle treatment, were merged according to their genotypes for the subsequent analysis. WT – wild type mice; FUS – FUS-tg mice; Veh – housing on tap water; Veh-icv – infusion of vehicle in i.c.v.; NC-icv – infusion of Neuro-Cells to i.c.v.

**Figure 5**

***Figure S5. Body weight at Weeks 1-6 of the experiment*.** Two-way ANOVA and post-hoc Tukey’s test revealed a significant effect of genotype on body weight, normalized to the means of control mice, at week 3 (F_3,29_=12.94, p=0.012, two-way ANOVA), week 4 (F_3,29_=28.11, p=0.002, two-way ANOVA), week 5 (F_3,29_=16.17, p<0.001, two-way ANOVA) week 6 (F_3,29_=19.35, p > 0.001, two-way ANOVA). In addition, there was a significant effect of riluzole treatment at week 6 (F_1,12_=14.26, p=0.039, two-way ANOVA), celecoxib treatment at week 5 (F_1,14_=8.13, p=0.017, two-way ANOVA) and week 6 (F_1,14_=5.63, p<0.001, two-way ANOVA), and Neuro-Cells treatment at week 4 (F_1,13_=12.85, p=0.033, two-way ANOVA), week 5 (F_1,13_=21.88, p=0.024, two-way ANOVA) and week 6 (F_1,13_=11.63, p=0.002, two-way ANOVA). Post-hoc analysis revealed a significant decrease in body weight in FUS-tg-Veh group at the week 3 (p=0.031, Tukey's test), week 4 (p=0.026, Tukey's test), week 5 (p=0.018, Tukey's test) and week 6 (p=0.001, Tukey's test), in comparison with wild type control mice. FUS-tg mice treated with riluzole showed a significant weight loss at week 6 (p=0.043, Tukey's test), and FUS-tg mice treated with celecoxib had this effect at weeks 5 and 6 (p=0.025 and p=0.019, respectively, Tukey's test) in comparison with wild type control mice.*p<0.05 vs. WT-Veh group. Thus, all treatments delayed a decrease of body weight in FUS-tg mice in comparison with controls, and infusion of Neuro-Cells preserved normal body weight longer than administration of celecoxib or riluzole.

**Figure 6**

**
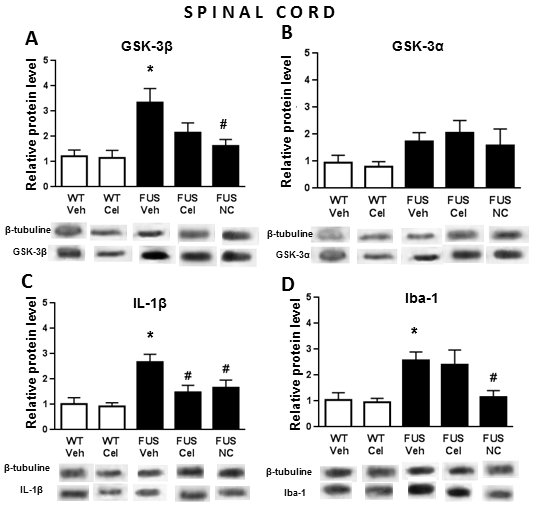
**

**Figure 6. Effects of administration of celecoxib and Neuro-Cells to FUS-tg mice on expression of molecular markers of inflammation and GSK-3.** Following examination of Western Blot bands we found significantly elevated protein levels of (**A**) GSK-3β, (**B**) IL-1β and (**C**) IL-1β and (**D**) Iba-1 in spinal cords of FUS-tg-Veh animals in comparison with WT-Veh mice that was not found in FUS-tg-Cel and FUS-tg-NC groups (p>0.05). (**D**) No significant differences were found in the protein levels of GSK-3α (p>0.05). Animals from FUS-tg-NC group showed significantly lower values of protein expression of GSK-3β, IL-1β and Iba-1 than FUS-tg mice treated with vehicle. Mice from FUS-tg-Cel group showed a significant decrease of protein expression of IL-1β in comparison with FUS-Veh group. β-tubulin was used as a loading control in the Western blot assay, the expression of all proteins is normalized to β-tubulin expression (*p<0.05, vs. WT-Veh group, #p<0.05, vs. FUS-tg-Veh group: one-way ANOVA and Tukey’s test), bars are Mean ± SEM. Data used for quantification are presented in Appendix 1.

**Reference list**

1. Donders R, Vanheusden M, Bogie JF, Ravanidis S, Thewissen K, Stinissen P, Gyselaers W, Hendriks JJ, Hellings N. [Human Wharton's Jelly-Derived Stem Cells Display immunomodulatory Properties and Transiently Improve Rat Experimental Autoimmune Encephalomyelitis.](https://www.ncbi.nlm.nih.gov/pubmed/25310756) Cell Transplant 2015;24:2077-98.
2. Gorlova A, Pavlov D, Anthony DC, Ponomarev ED, Sambon M, Proshin A, Shafarevich I, Babaevskaya D, Lesсh KP, Bettendorff L, Strekalova T. [Thiamine and benfotiamine counteract ultrasound-induced aggression, normalize AMPA receptor expression and plasticity markers, and reduce oxidative stress in mice.](https://www.ncbi.nlm.nih.gov/pubmed/30817932) Neuropharmacology 2019;156:107543.
3. Kovina MV, Karnaukhov AV, Krasheninnikov ME, Kovin AL, Gazheev ST, Sergievich LA, Karnaukhova EV, Bogdanenko EV, Balyasin MV, Khodarovich YM, Dyuzheva TG, Lyundup AV. [Extension of Maximal Lifespan and High Bone Marrow Chimerism After Nonmyeloablative Syngeneic Transplantation of Bone Marrow From Young to Old Mice.](https://www.ncbi.nlm.nih.gov/pubmed/31031800) Front Genet 2019;10:310.
4. Pavlov D, Bettendorff L, Gorlova A, Olkhovik A, Kalueff AV, Ponomarev ED, Inozemtsev A, Chekhonin V, Lesсh KP, Anthony DC, Strekalova T. [Neuroinflammation and aberrant hippocampal plasticity in a mouse model of emotional stress evoked by exposure to ultrasound of alternating frequencies.](https://www.ncbi.nlm.nih.gov/pubmed/30472146) Prog Neuropsychopharmacol Biol Psychiatry 2019;90:104-116.
