# Supplementary material for: Neuro‐Cells therapy improves motor outcomes and suppresses inflammation during experimental syndrome of amyotrophic lateral sclerosis in mice
Source: CNS Neurosci Ther. 2019 Dec 23;26(5):504–17. doi: 10.1111/cns.13280 (PMC7163689; doi:10.1111/cns.13280)

GSK3b: 46kDa

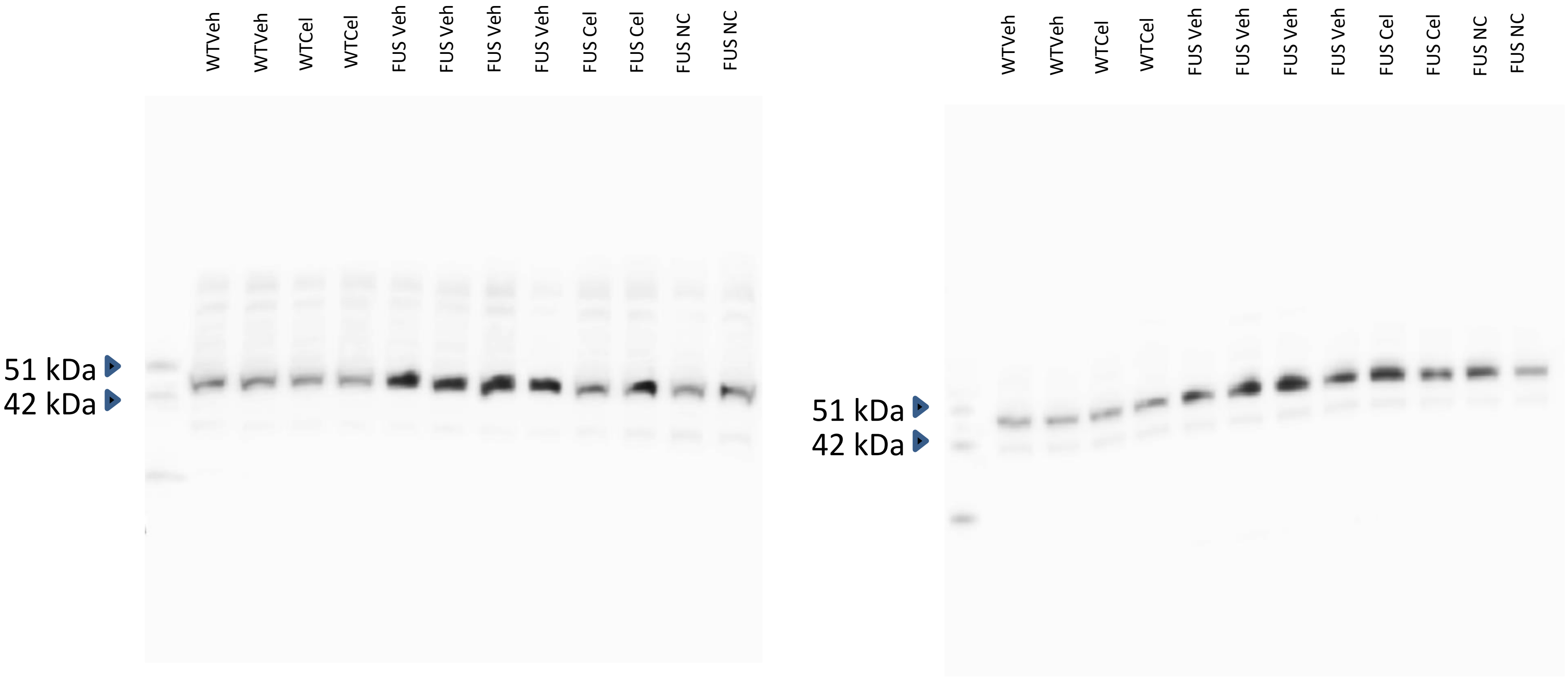
WTVeh
WTVeh
WTCel
WTCel
FUS Veh
FUS Veh
FUS Veh
FUS Veh
FUS Cel
FUS Cel
FUS NC
FUS NC
51 kDa
42 kDa
WTVeh
WTVeh
WTCel
WTCel
FUS Veh
FUS Veh
FUS Veh
FUS Veh
FUS Cel
FUS Cel
FUS NC
FUS NC
51 kDa
42 kDa

GSK3a: 51kDa

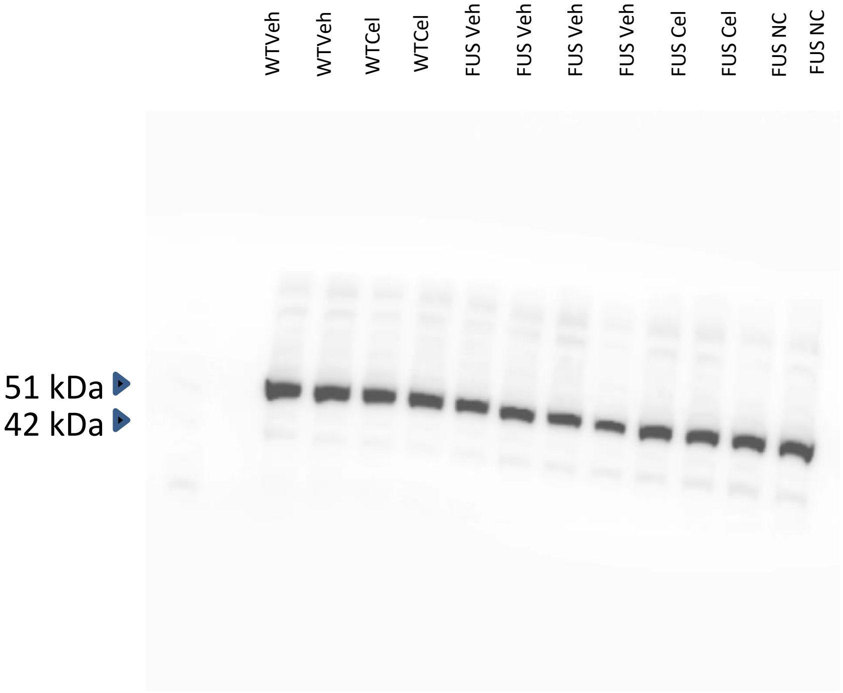
WTVeh
WTVeh
WTCel
WTCel
FUS Veh
FUS Veh
FUS Veh
FUS Veh
FUS Cel
FUS Cel
FUS NC
FUS NC
51 kDa
42 kDa

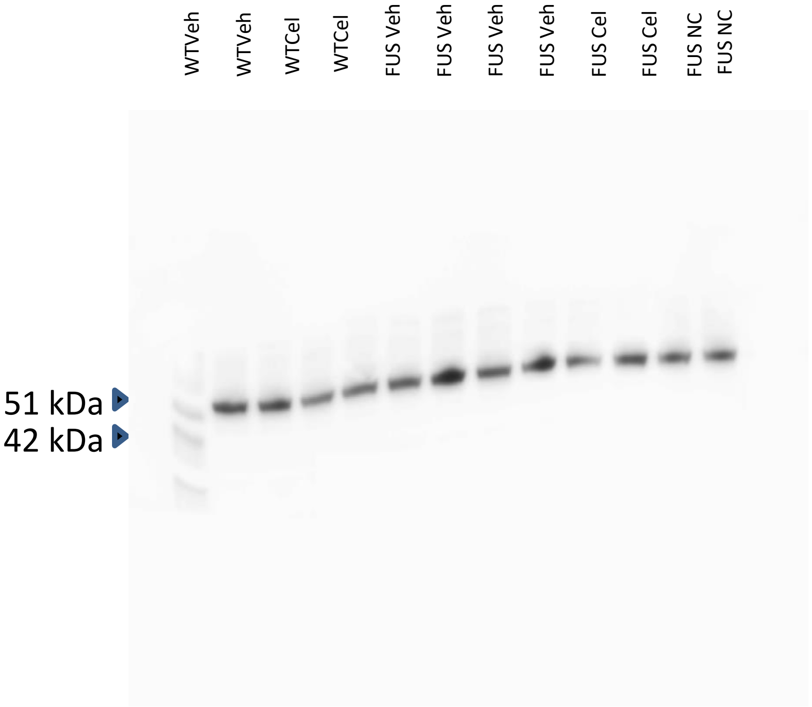
WTVeh
WTVeh
WTCel
WTCel
FUS Veh
FUS Veh
FUS Veh
FUS Veh
FUS Cel
FUS Cel
FUS NC
FUS NC
51 kDa
42 kDa

IL1b: 17kDa

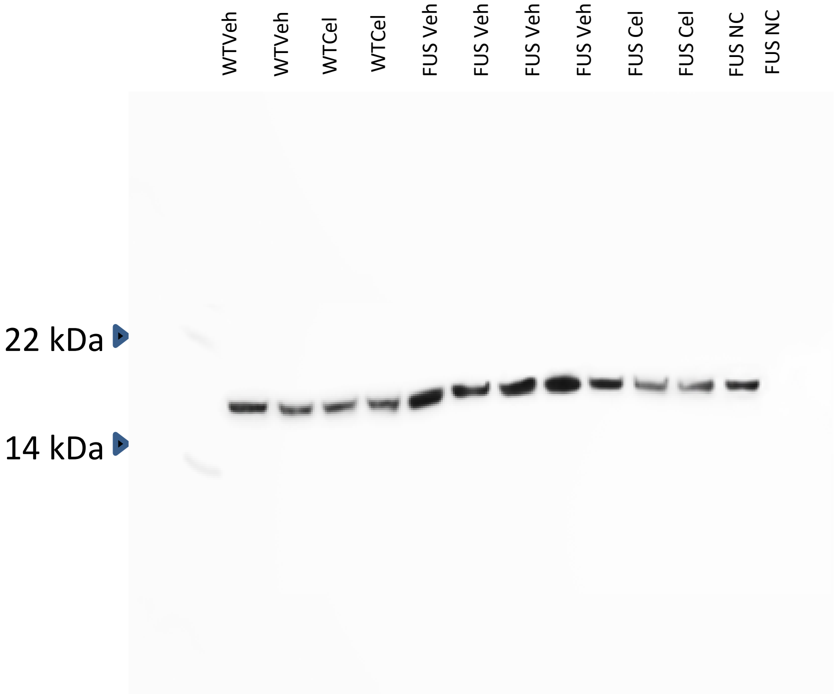
WTVeh
WTVeh
WTCel
WTCel
FUS Veh
FUS Veh
FUS Veh
FUS Veh
FUS Cel
FUS Cel
FUS NC
FUS NC
22 kDa
14 kDa

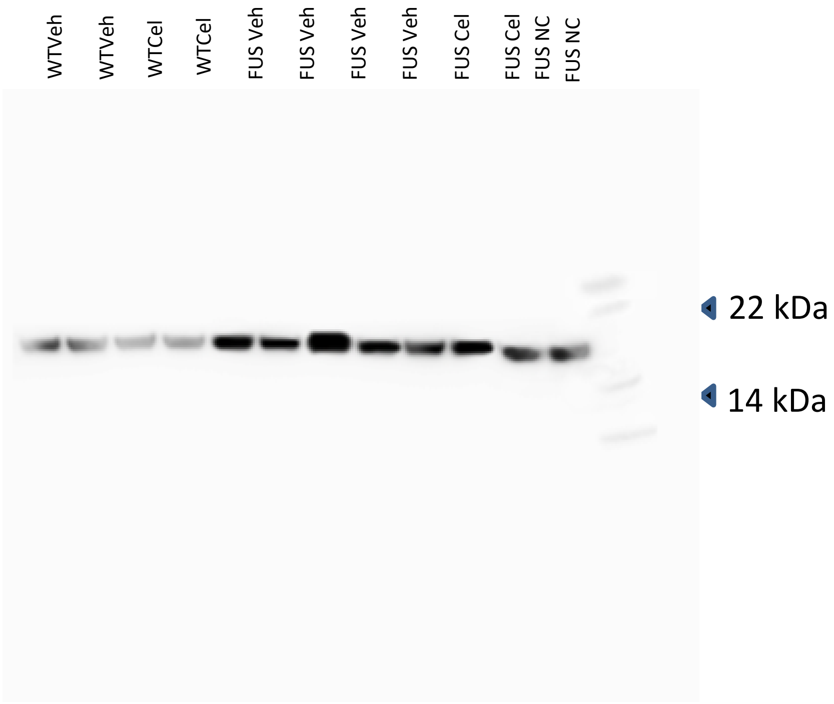
WTVeh
WTVeh
WTCel
WTCel
FUS Veh
FUS Veh
FUS Veh
FUS Veh
FUS Cel
FUS Cel
FUS NC
FUS NC
22 kDa
14 kDa

Iba1: 17kDa

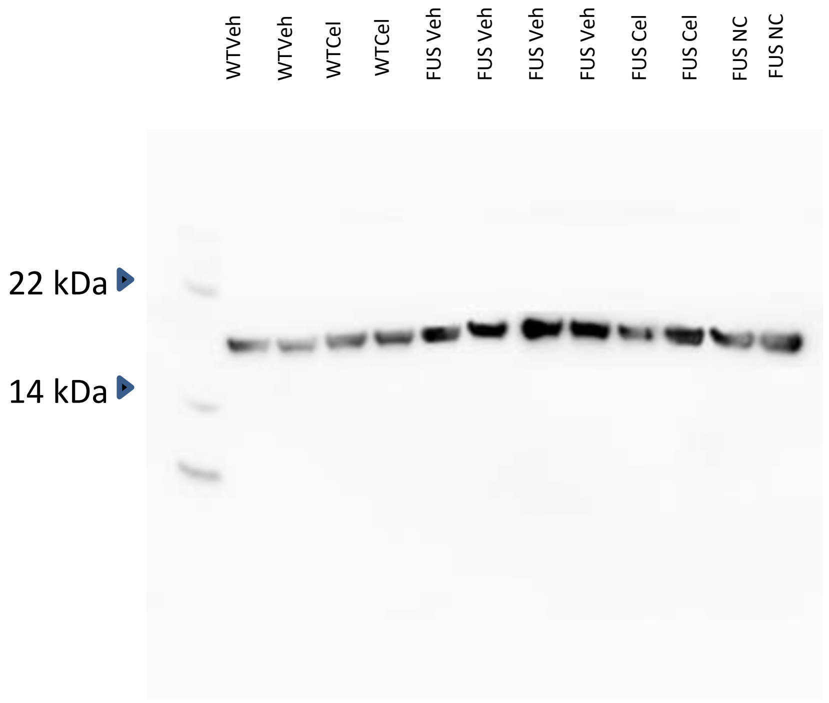
WTVeh
WTVeh
WTCel
WTCel
FUS Veh
FUS Veh
FUS Veh
FUS Veh
FUS Cel
FUS Cel
FUS NC
FUS NC
22 kDa
14 kDa

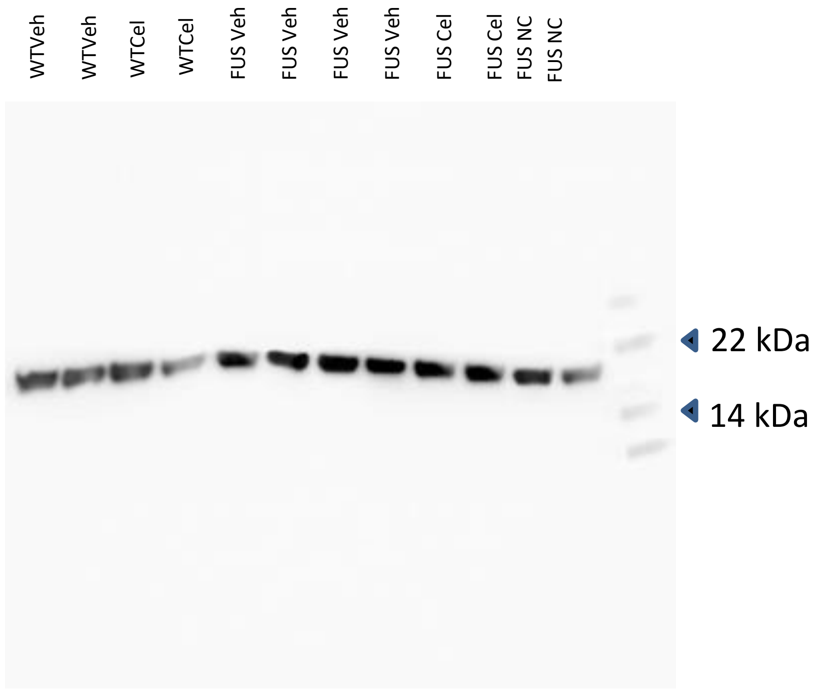
WTVeh
WTVeh
WTCel
WTCel
FUS Veh
FUS Veh
FUS Veh
FUS Veh
FUS Cel
FUS Cel
FUS NC
FUS NC
22 kDa
14 kDa

B Tubuline: 55kDa

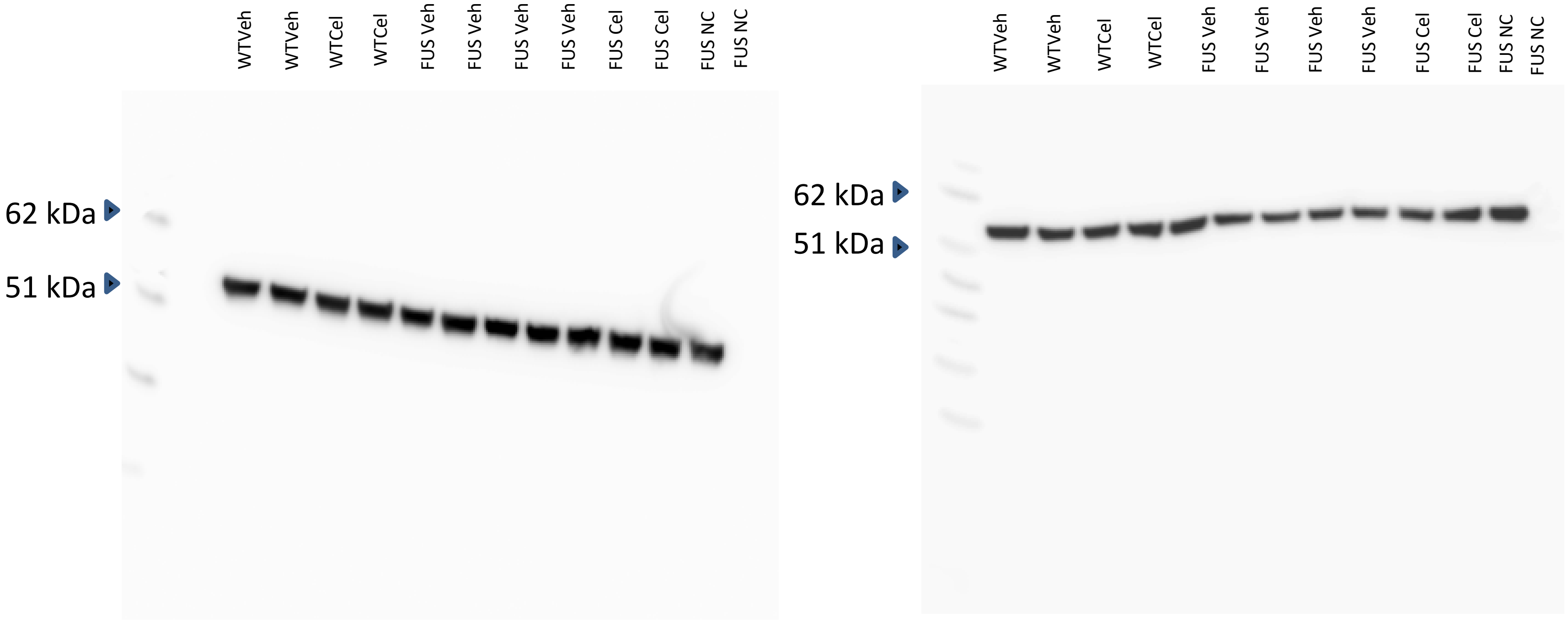

B Tubuline: 55kDa

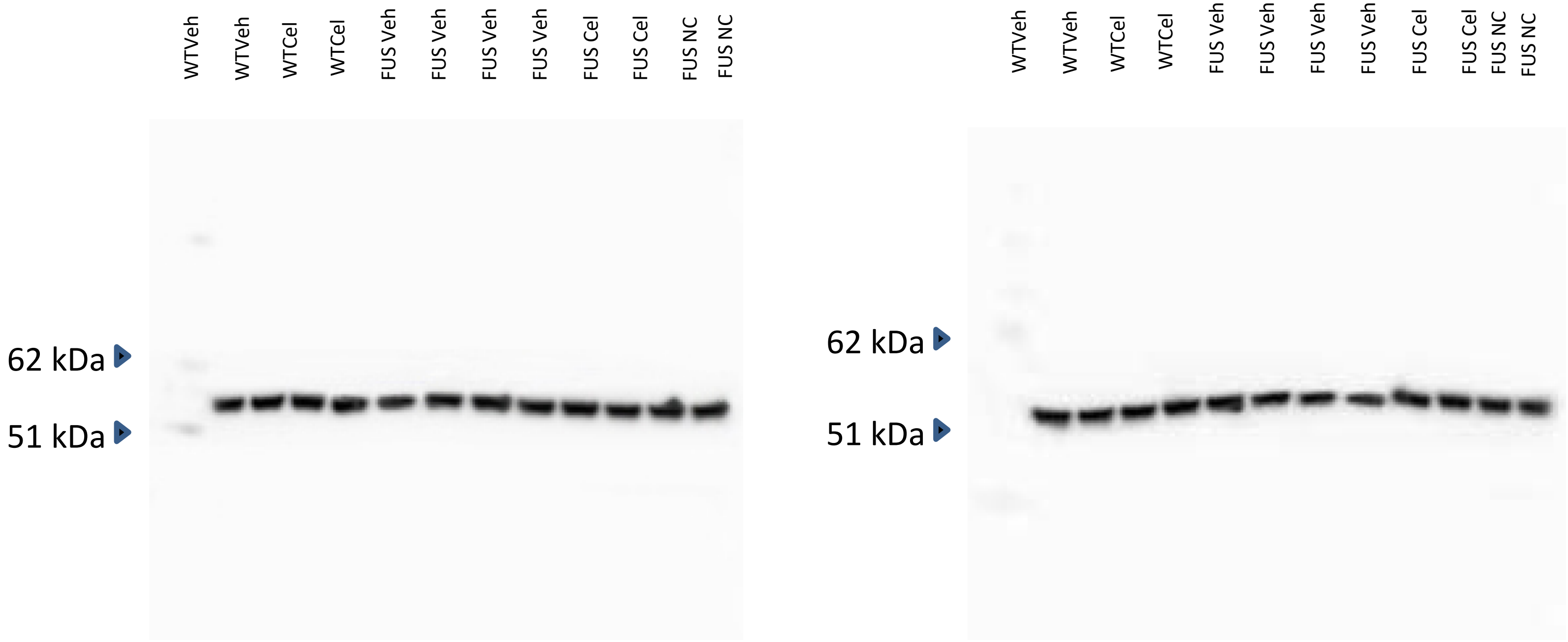

B Tubuline: 55kDa

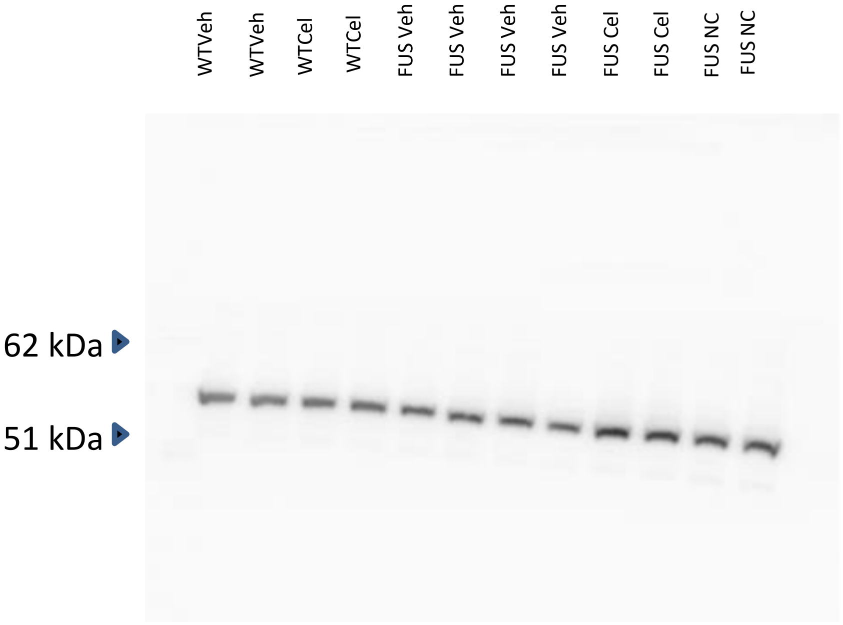

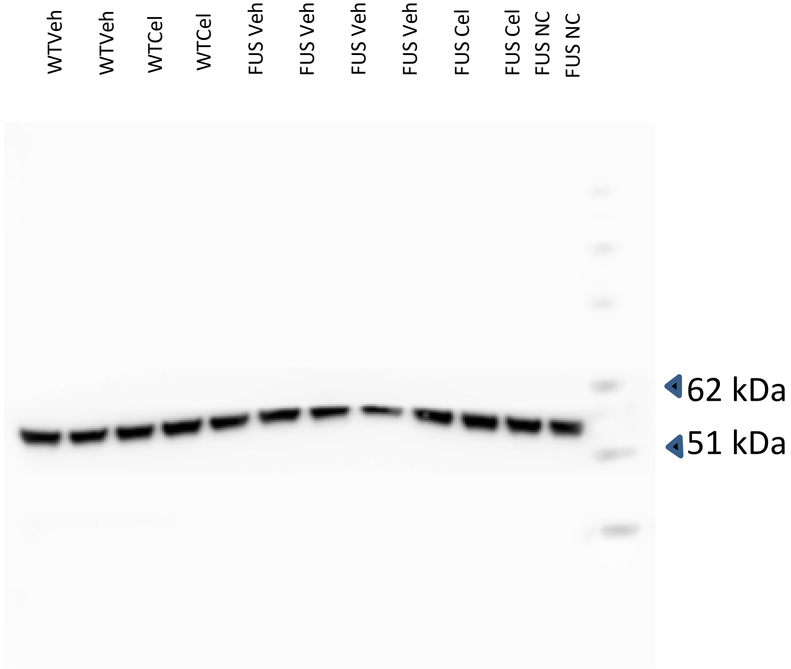

B Tubuline: 55kDa

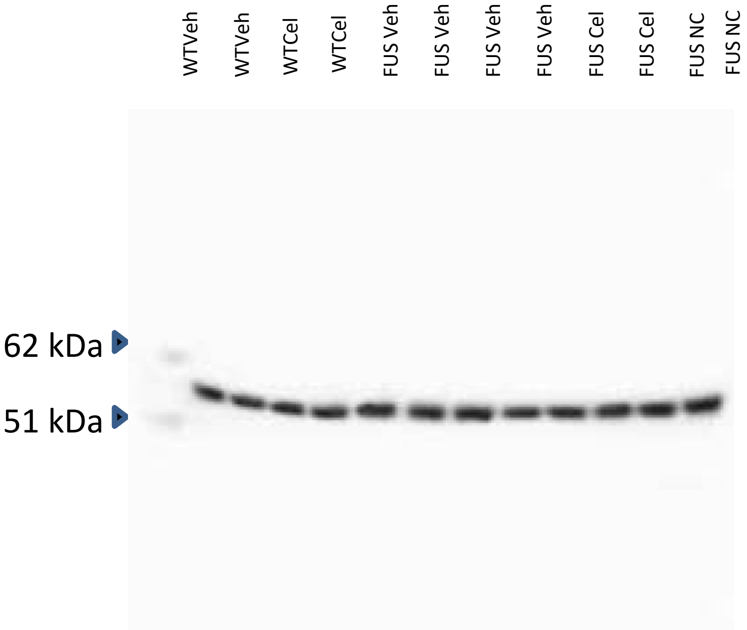

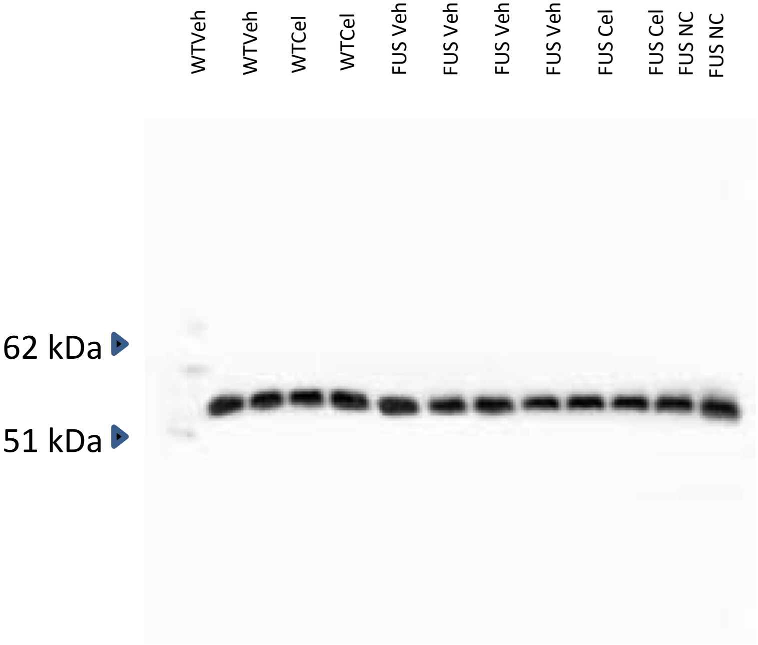

Supplement: Supplementary file 2 [file CNS-26-504-s002.pdf]
